# Genome‐Resolved Insights Into Phosphate‐Solubilizing Bacteria From Wild Rice Rhizosphere Reveal Adaptive Strategies for Phosphorus Cycling Under Acid‐Saline Stress in the Mekong Delta, Vietnam

**DOI:** 10.1002/ece3.74327

**Published:** 2026-09-23

**Authors:** Xuyen Thi Hong Nguyen, Tan Khang Do, Giang Thi Tran

**Affiliations:** ^1^ Institute of Food and Biotechnology Can Tho University Can Tho Vietnam; ^2^ Faculty of Food and Biochemical Technology Can Tho University of Technology Can Tho Vietnam; ^3^ The Biotech‐CTU Research Group Can Tho University Can Tho Vietnam

**Keywords:** acid tolerance, *Enterobacter hormaechei*, genome sequencing, phosphate solubilization, wild rice ( *Oryza rufipogon* )

## Abstract

Phosphorus (P) limitation constrains crop productivity in acid and saline soils of the Mekong Delta, where low P availability rather than total P content limits plant growth. This study aimed to isolate phosphate‐solubilizing bacteria (PSB) from the rhizosphere of wild rice ( *Oryza rufipogon* ) and to investigate their functional traits and genomic basis of adaptation. A total of 43 bacterial isolates were obtained, of which 10 showed strong phosphate‐solubilizing activity (PSI 1.5–2.5). Selected strains released up to ~900 mg L^−1^ soluble phosphorus after 10 days, with strain GC1.3 exhibiting high tolerance to pH 4–7 and 0.1%–1.0% NaCl, and exceeding 1000 mg L^−1^ at pH 6. Whole‐genome sequencing revealed that GC1.3 belongs to *Enterobacter hormaechei* (ANI = 95.93%) with a complete genome of 4.78 Mb. Genomic analysis identified key genes involved in phosphate mobilization, including *gcd* and *pqqC* (organic acid production), *phnC‐phnD* (phosphonate uptake), and *ppk‐ppx* (polyphosphate metabolism), along with multiple stress‐response systems related to ion homeostasis, osmoprotection, and oxidative stress. Notably, GC1.3 harbors 411 phosphate‐related genes, exceeding those of reference strains. These findings demonstrate that wild rice rhizosphere harbors metabolically versatile PSB capable of sustaining phosphorus cycling under acid‐saline stress. Strain *E. hormaechei* GC1.3 shows strong potential as a biofertilizer candidate for improving P availability in stress‐prone agricultural soils.

## Graphical abstract

This study aimed to isolate phosphate‐solubilizing bacteria (PSB) from the rhizosphere of wild rice ( *Oryza rufipogon* ) and to investigate their functional traits and genomic basis of adaptation. Genomic analysis of *Enterobacter hormaechei* GC1.3 identified key genes involved in phosphate mobilization, including *gcd* and *pqqC* (organic acid production), *phnC*‐*phnD* (phosphonate uptake), and *ppk*‐*ppx* (polyphosphate metabolism), along with multiple stress‐response systems related to ion homeostasis, osmoprotection, and oxidative stress. Notably, GC1.3 harbors 411 phosphate‐related genes, exceeding those of reference strains. These findings demonstrate that wild rice rhizosphere harbors metabolically versatile PSB capable of sustaining phosphorus cycling under acid‐saline stress.

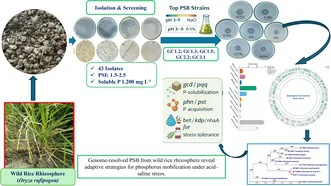

## 1 Introduction

Phosphorus (P) is an essential macronutrient that plays a central role in plant growth, energy transfer, and metabolic regulation. However, in most agricultural soils, phosphorus availability is limited due to its fixation in poorly soluble inorganic forms or incorporation into organic complexes, resulting in low bioavailability despite relatively high total P content. Consequently, phosphorus deficiency remains a major constraint on crop productivity worldwide (Behera et al. 2014; Tian et al. 2021).

Phosphate‐solubilizing bacteria (PSB) are key components of the soil microbiome that contribute to phosphorus cycling by converting insoluble phosphate into plant‐available forms. This process is primarily mediated through the secretion of organic acids, such as gluconic, citric, and oxalic acids, which lower the pH and chelate metal ions including Ca^2+^, Fe^3+^, and Al^3+^, thereby releasing soluble phosphate (Rodríguez and Fraga 1999; Alori et al. 2017). In addition to mineral phosphate solubilization, PSB also participate in broader biogeochemical processes, including organic phosphorus mineralization and nutrient mobilization, highlighting their ecological importance in maintaining soil fertility (Timofeeva et al. 2022; Li et al. 2023).

The ecological role of PSB becomes particularly critical in stress‐prone environments. In the Mekong Delta, agricultural soils are increasingly affected by acid sulfate conditions and salinity intrusion, both of which significantly reduce phosphorus availability and microbial activity. These harsh conditions impose strong selective pressures on soil microbial communities, leading to the enrichment of stress‐adapted microorganisms capable of maintaining functional activity under fluctuating pH and salinity regimes (Wang et al. 2025; Egamberdieva et al. 2019). In this context, the rhizosphere of wild rice ( *Oryza rufipogon* ) represents a unique ecological niche where long‐term environmental selection pressures associated with acidic and saline conditions have favored microbial communities possessing adaptive traits for nutrient acquisition and stress tolerance.

Recent advances in microbial genomics have provided new opportunities to link functional traits with underlying genetic mechanisms. Phosphate solubilization is now recognized as a multifaceted process involving coordinated pathways for organic acid production, phosphate transport, and intracellular phosphorus storage and remobilization (Pan and Cai 2023). In particular, the pyrroloquinoline quinone (PQQ)‐dependent glucose dehydrogenase system plays a dominant role in inorganic phosphate solubilization in Gram‐negative bacteria by facilitating gluconic acid production (Bhanja et al. 2021). In addition, genes associated with phosphonate uptake (phn operon), polyphosphate metabolism (*ppk*–*ppx*), and high‐affinity phosphate transport systems (*pstSCAB*) contribute to efficient phosphorus acquisition under nutrient‐limited conditions. However, despite increasing interest in PSB, the integration of physiological performance with genome‐level functional analysis remains limited, particularly for plant‐associated bacteria inhabiting environments simultaneously affected by salinity and acidity (Raturi et al. 2022; Escalante‐Beltrán et al. 2025).

Among plant‐associated bacteria, *E. hormaechei* has emerged as a promising plant growth‐promoting rhizobacterium because of its metabolic versatility and ecological adaptability. Salt‐tolerant strains of *E. hormaechei* have been shown to retain key plant growth‐promoting traits, including phosphate solubilization, indole‐3‐acetic acid production, rhizosphere colonization, and stimulation of plant growth under saline conditions, demonstrating their capacity to remain metabolically active in environmentally stressed soils (Egamberdieva et al. 2014; Ranawat et al. 2021; Roslan et al. 2020). Comparative genomic analyses of plant‐associated *Enterobacter* spp. further revealed diverse functional genes involved in nutrient acquisition and transport, chemotaxis, membrane transport, oxidative stress defense, regulatory networks, and plant colonization, providing a genomic basis for adaptation to fluctuating environmental conditions while sustaining beneficial plant‐microbe interactions (Andrés‐barrao et al. 2023). Recent genome sequencing studies have also shown that *E. hormaechei* harbors numerous genes associated with plant growth promotion and environmental stress tolerance, highlighting the value of genome‐based analyses for understanding the genetic mechanisms underlying rhizosphere fitness and ecological adaptation (Ahmed et al. 2026). Although the molecular mechanisms of acid tolerance have not yet been comprehensively characterized in *E. hormaechei* , members of the family Enterobacteriaceae generally employ conserved acid‐resistance systems, including intracellular pH homeostasis, amino acid‐dependent acid resistance pathways, and global stress‐response regulators, which enable survival under low‐pH conditions (Foster 2004). Together, these physiological traits and conserved molecular mechanisms suggest that stress‐adapted *Enterobacter* strains are well suited for biofertilizer development in agricultural soils simultaneously affected by salinity and acidity.

Therefore, the present study aimed to isolate and characterize phosphate‐solubilizing bacteria from the rhizosphere of wild rice in the Mekong Delta, Vietnam, and to evaluate their functional performance under acid and saline conditions. Furthermore, whole‐genome sequencing and functional annotation were conducted for the most promising isolate to elucidate the genetic determinants associated with phosphorus mobilization and environmental adaptation. By integrating physiological assays with genome‐based analysis, this study provides ecological insights into microbial adaptation in stress‐prone agroecosystems and contributes to the development of sustainable biofertilizer strategies for improving phosphorus use efficiency.

## 2 Materials and Methods

### 2.1 Isolation and Morphological Characterization of Phosphate‐Solubilizing Bacteria

Rhizosphere soil samples were collected from wild rice ( *O. rufipogon* ) populations at eight sampling locations in Go Cong Dong and Cai Lay districts, Tien Giang Province, Vietnam, in October 2024. Wild rice plants were carefully uprooted together with the surrounding soil. Loosely attached soil was gently removed by shaking, and only the soil tightly adhering to the root surface was collected as rhizosphere soil for bacterial isolation. At each location, rhizosphere soil adhering to the roots of the sampled wild rice plants was pooled to form one composite sample, and each sampling location was considered one biologically independent sample. Geographic coordinates and detailed administrative information are provided in Table S1. Samples were transported to the laboratory under refrigerated conditions and processed within 24 h of collection.

Rhizosphere soil samples were collected from wild rice ( *O. rufipogon* ) in the Mekong Delta, Vietnam. Soil suspensions were serially diluted and plated on National Botanical Research Institute's phosphate (NBRIP) agar containing tricalcium phosphate (Ca_3_(PO_4_)_2_) as the sole phosphorus source (Nautiyal 1999). Plates were incubated at 30°C ± 2°C for 5–7 days. Colonies showing clear halo zones were selected as PSB, purified by repeated streaking, and maintained on nutrient agar at 4°C. Colony morphology (shape, color, and surface characteristics) was recorded. Basic phenotypic characterization of selected isolates included Gram staining, catalase activity, and motility using standard microbiological methods (Cappuccino and Welsh 2019).

### 2.2 Qualitative Screening of PSB

Isolates were cultured in LB medium (Bertani 1951) at 30°C ± 2°C with shaking (150 rpm) for 12 h to reach OD_600_ ≈0.8 (~10^8^ cells mL^−1^). A 10 μL aliquot was spot‐inoculated onto NBRIP agar and incubated for 5–7 days. Colony and halo diameters were measured, and the phosphate solubilization index (PSI) was calculated as follows: PSI = (colony diameter + halo diameter)/colony diameter (Afzal and Bano 2008). All experiments were conducted in triplicate.

### 2.3 Quantitative Phosphate Solubilization Assay

A 0.5 mL aliquot of bacterial culture (OD_600_ ≈0.8) was inoculated into 20 mL of NBRIP liquid medium, and the cultures were incubated at 30°C ± 2°C with shaking at 150 rpm for 5, 10, and 15 days. Non‐inoculated medium served as a control. Cultures were centrifuged (8000 rpm, 10 min). Soluble phosphate was quantified using the Murphy and Riley colorimetric method. A calibration curve was prepared using potassium dihydrogen phosphate (KH_2_PO_4_) standard solutions at known phosphate concentrations (Murphy and Riley 1962). Results were expressed as mg L^−1^ (mean ± SD, *n* = 3).

### 2.4 Effects of Salinity and pH

Five representative strains were evaluated under different NaCl concentrations (0%–1.0%) and pH levels (3–9) in NBRIP broth. Cultures were incubated under the same conditions as described above and sampled at 5, 10, and 15 days. Bacterial growth was measured as OD_600_, and soluble phosphorus was quantified as described in Section 2.3. Experiments followed a completely randomized design with three replicates.

NaCl was selected as the salinity source because seawater intrusion is the primary cause of salinization in the Mekong Delta, where Na^+^ and Cl^−^ are the dominant ions. Therefore, NaCl was considered the most environmentally relevant salt for evaluating the salinity tolerance of phosphate‐solubilizing bacteria intended for agricultural application in this region.

### 2.5 Molecular Identification (16S rRNA)

Genomic DNA was extracted from freshly cultured bacterial cells using an in‐house SDS–ethanol precipitation method, and DNA concentration and purity were assessed using a NanoDrop spectrophotometer (Thermo Fisher Scientific, USA). The nearly full‐length 16S rRNA gene was amplified using the universal primers 27F (5′‐AGAGTTTGATCMTGGCTCAG‐3′) and 1492R (5′‐TACGGYTACCTTGTTACGACTT‐3′), yielding an approximately 1500‐bp amplicon (Weisburg et al. 1991). PCR amplification was performed in a 25‐μL reaction under the conditions described above. Amplicons were verified by agarose gel electrophoresis and bidirectionally sequenced using the Sanger method on an ABI 3130xl Genetic Analyzer (Applied Biosystems, USA). Forward and reverse chromatograms were quality‐checked, trimmed, and assembled into consensus sequences using BioEdit v7.2.1. Preliminary taxonomic identification was performed using BLASTn against the NCBI nucleotide database (Altschul et al. 1990). Closely related reference sequences were aligned using ClustalW, and phylogenetic relationships were inferred by the Maximum Likelihood method under the HKY + I substitution model with 1000 bootstrap replicates in MEGA12 (Kumar et al. 2024). Strain identification was based on both sequence similarity and phylogenetic placement.

Among the identified strains, GC1.3 was selected for whole‐genome sequencing based on its overall performance in phosphate solubilization, tolerance to salinity and pH stress, and its representativeness of isolates from the saline‐affected region of the Mekong Delta.

### 2.6 Whole‐Genome Sequencing and Bioinformatics Analysis

The bacterial isolate GC1.3 was cultured in LB medium and subjected to whole‐genome sequencing using a hybrid approach combining short‐read (Illumina) (Illumina 2011) and long‐read (Oxford Nanopore) data to improve assembly accuracy and completeness (Wick et al. 2017; Krasnov et al. 2020).

Raw reads were quality‐filtered using fastp (Chen et al. 2018), and data quality was assessed based on standard metrics, including read number, total bases, read length, GC content, and quality scores (%Q30 for short reads and > Q7 for long reads). Cleaned reads were assembled de novo using Unicycler (Wick et al. 2017). Assembly quality was evaluated using QUAST (Gurevich et al. 2013) and CheckM v1.2.4 (Parks et al. 2015). Genome completeness was assessed using BUSCO v5.8.0 in protein mode with the Enterobacterales‐specific lineage dataset enterobacterales_odb10, comprising 440 conserved single‐copy orthologs (Manni et al. 2021).

Gene prediction and annotation were performed using Prokka and Prodigal (Seemann 2014). Taxonomic assignment was conducted using GTDB‐Tk (Chaumeil et al. 2020). Functional annotation was carried out using eggNOG‐mapper (Cantalapiedra et al. 2021), and KEGG Orthology (KO) terms were assigned using KofamKOALA (Aramaki et al. 2020) for pathway reconstruction (Kanehisa et al. 2025). All analyses were performed on the Galaxy Europe platform (The Galaxy Community 2024).

### 2.7 Statistical Analysis

All experiments were conducted in triplicate. Data were expressed as mean ± standard deviation. Statistical differences among treatments were determined using one‐way ANOVA followed by Fisher's multiple range test at *p* < 0.05. Data analysis and visualization were performed using Minitab 19 and Microsoft Excel.

## 3 Results

### 3.1 Isolation and Morphological Diversity of Bacterial Strains

A total of 43 bacterial isolates obtained from the rhizosphere soil of wild rice ( *O. rufipogon* ) in the Mekong Delta, Vietnam were characterized based on colony morphology, cell morphology, and Gram‐staining properties (Table 1). Colony morphology showed noticeable variability in shape, color, margin, surface, and elevation. Most colonies were circular, while a smaller proportion displayed irregular or punctiform shapes. Microscopic observation revealed that rod‐shaped bacteria were slightly more abundant than cocci. Out of the 43 isolates, 19 strains (44.18%) exhibited rod‐shaped cells, whereas 24 strains (55.81%) were cocci. Gram‐staining results indicated that the majority of isolates were Gram‐negative. Specifically, 41 strains (95.34%) were Gram‐negative, while only 2 strains (4.65%) were Gram‐positive. These results indicate that Gram‐negative bacteria predominated among the isolates obtained from the wild rice rhizosphere in the Mekong Delta, Vietnam.

**TABLE 1:** Morphological characteristics of bacterial isolates from wild rice rhizosphere soil.

No.	Strains code	Colony morphology (shape, color, margin, surface, elevation)	Cell morphology	Gram staining	Catalase test	Mobility test
1	GC1.1	Circular, transparent white, entire, smooth, raised	Coccus	(−)	−	+
2	GC1.2	Circular, transparent white, entire, glistening, flat	Rod	(−)	+	+
3	GC1.3	Circular transparent white, undulate, smooth, flat	Rod	(−)	+	+
4	GC1.4	Circular, opaque yellow, undulate, wrinkled, convex	Rod	(−)	+	+
5	GC1.5	Circular, transparent white, entire, smooth, flat	Rod	(−)	+	−
6	GC1.6	Punctiform, opaque white, entire, wrinkled, flat	Coccus	(−)	+	+
7	GC2.1	Circular, transparent white, smooth, raised	Coccus	(−)	−	+
8	GC2.2	Circular, opaque white, entire, smooth, convex	Rod	(−)	+	+
9	GC2.3	Circular, opaque white, undulate, smooth, flat	Coccus	(−)	−	+
10	GC2.4	Circular, opaque white, entire, dry, flat	Coccus	(−)	+	+
11	GC3.1	Circular, opaque white, entire, smooth, raised	Coccus	(−)	+	−
12	GC3.2	Circular, transparent yellow, entire, glistening, raised	Coccus	(−)	+	+
13	GC3.3	Circular, opaque white, undulate, smooth, raised	Coccus	(−)	+	+
14	GC3.4	Circular, opaque white, undulate, smooth, flat	Rod	(−)	+	−
15	GC4.1	Circular, transparent white, undulate, dry, flat	Coccus	(−)	+	+
16	GC4.2	Circular, opaque white, undulate, smooth, raised	Coccus	(−)	−	+
17	GC4.3	Punctiform, transparent white, entire, smooth, flat	Coccus	(−)	−	+
18	GC4.4	Punctiform, opaque white, entire, smooth, flat	Coccus	(−)	−	+
19	GC4.5	Circular, opaque yellow, undulate, wrinkled, raised	Coccus	(−)	−	+
20	CL1.1	Irregularly circular, translucent white, undulate, smooth, convex	Rod	(−)	−	+
21	CL1.3	Circular, translucent white, entire, smooth, convex	Rod	(−)	+	+
22	CL1.4	Irregularly circular, opaque white, entire, smooth, convex	Coccus	(−)	−	+
23	CL1.5	Irregularly circular, translucent white, entire, smooth, convex	Coccus	(−)	+	+
24	CL1.6	Irregularly circular, translucent white, entire, smooth, convex	Coccus	(−)	−	+
25	CL2.1	Circular, translucent white, entire, smooth, convex	Rod	(−)	−	+
26	CL2.2	Pinpoint, translucent white, entire, smooth, convex	Coccus	(−)	−	+
27	CL2.3	Circular, translucent white, entire, smooth, flat	Rod	(−)	+	+
28	CL2.4	Pinpoint, translucent white, entire, smooth, convex	Rod	(−)	+	+
29	CL2.5	Irregularly circular, translucent white, entire, smooth, convex	Coccus	(−)	−	+
30	CL2.6	Irregularly circular, opaque white, entire, rough, wrinkled	Coccus	(−)	−	+
31	CL3.1	Irregularly circular, translucent white, entire, dry, flat	Rod	(−)	−	+
32	CL3.2	Circular, translucent white, entire, smooth, flat	Coccus	(−)	−	+
33	CL3.3	Irregularly circular, opaque white, entire, smooth, flat	Rod	(−)	+	+
34	CL3.4	Circular, translucent white, entire, smooth, convex	Coccus	(−)	+	+
35	CL3.5	Irregularly circular, translucent white, entire, smooth, flat	Coccus	(−)	+	+
36	CL4.2	Pinpoint, translucent white, entire, smooth, flat	Coccus	(−)	−	+
37	CL4.3	Pinpoint, opaque white, entire, smooth, flat	Rod	(−)	−	+
38	CL4.4	Irregularly circular, opaque white, entire, smooth, flat	Rod	(−)	−	+
39	CL4.5	Pinpoint, opaque white, entire, smooth, flat	Rod	(−)	−	+
40	CL4.6	Pinpoint, opaque white, entire, smooth, flat	Rod	(−)	−	+
41	CL4.7	Pinpoint, opaque white, entire, smooth, flat	Rod	(−)	−	+
42	CL4.8	Circular, translucent white, entire, smooth, flat	Rod	(−)	−	+
43	CL4.9	Circular, translucent white, entire, smooth, flat	Coccus	(+)	+	+

*Note:* “+”, Positive; “−”, Negative.

### 3.2 Screening of PSB

**FIGURE 1:**
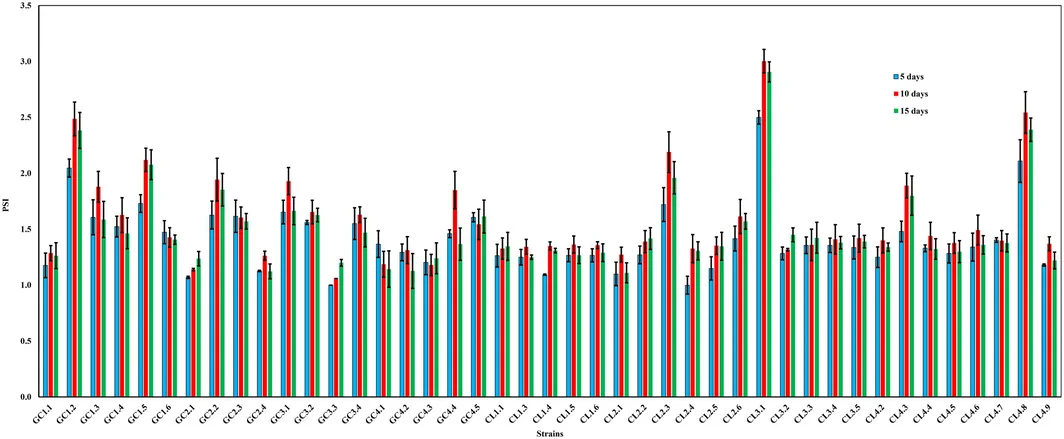
Phosphate solubilization index (PSI) at different incubation times. Values are presented as mean ± SD (*n* = 3). Statistical differences among strains at each sampling time were analyzed using one‐way ANOVA followed by Tukey's HSD test (*p* < 0.05).

The phosphate‐solubilization potential of the 43 bacterial isolates was evaluated by determining the phosphate solubilization index (PSI) after 5, 10, and 15 days of incubation. PSI values varied among isolates and across incubation periods, ranging from 1.00 to 3.00 (Figure 1). At day 10, most isolates exhibited PSI values between 1.0 and 2.0, whereas five isolates (CL3.1, CL4.8, GC1.2, CL2.3, and GC1.5) showed PSI values > 2.0. Among all isolates, strain CL3.1 exhibited the highest PSI (3.00 ± 0.10), followed by CL4.8 (2.54 ± 0.18) and GC1.2 (2.49 ± 0.15). Based on the PSI values obtained at day 10, the isolates were ranked from highest to lowest, and the 10 isolates with the highest PSI values were selected for subsequent quantitative phosphate‐solubilization assays. Overall, the PSI assay demonstrated variation in phosphate‐solubilization ability among the isolates and served as the primary screening criterion for selecting promising candidates for further evaluation. Detailed PSI values (mean ± SD) for all isolates at each incubation time are provided in Table S2.

**FIGURE 2:**
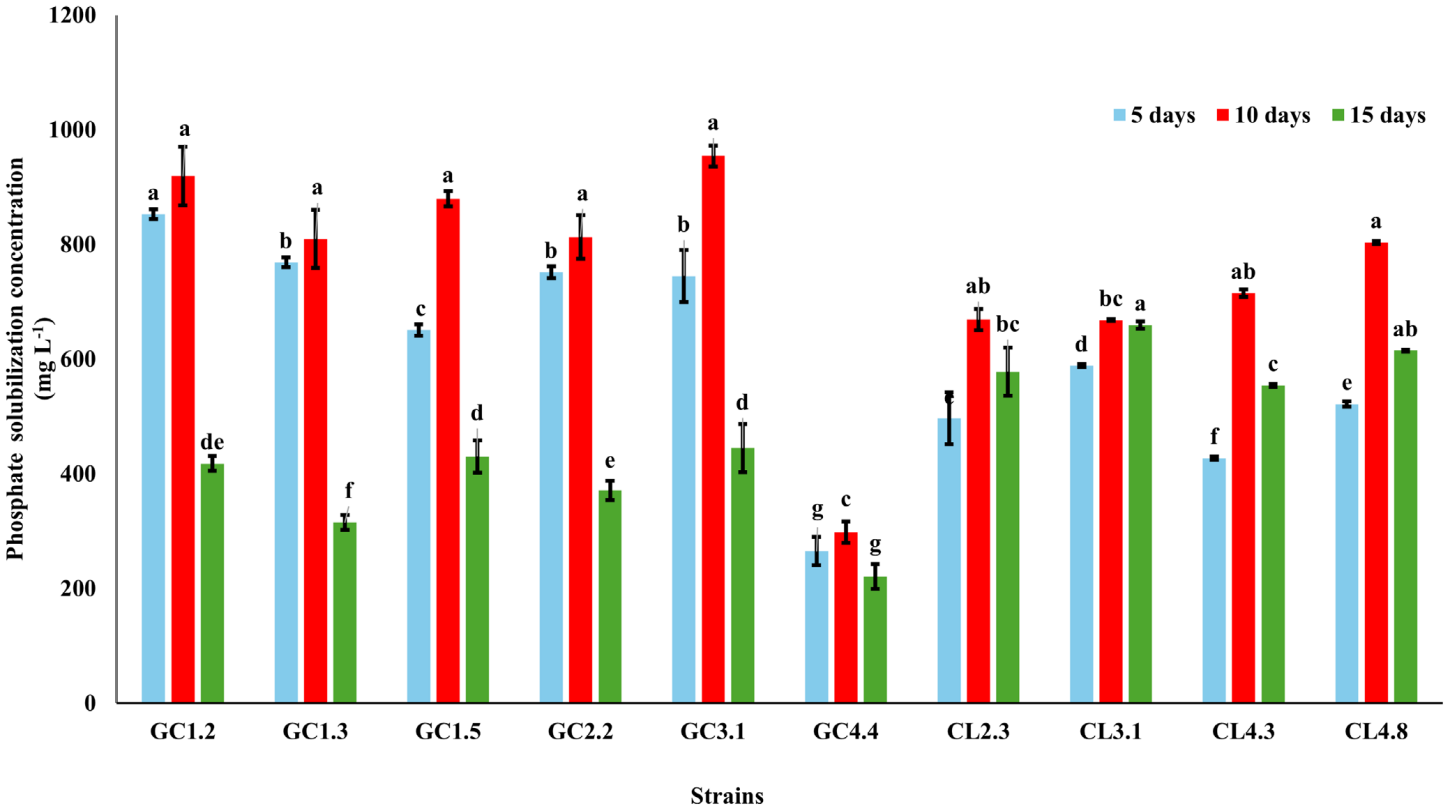
Soluble phosphorus concentration produced by bacterial isolates at different incubation times (5, 10, and 15 days). Values represent mean ± SD (*n* = 3). Different letters indicate significant differences (*p* < 0.05).

Based on the PSI values obtained at day 10, the 10 isolates with the highest PSI values were selected for quantitative phosphate‐solubilization assays in liquid NBRIP medium (Figure 2). Subsequently, the five isolates exhibiting the highest soluble phosphate concentrations were selected for further physiological characterization under different salinity and pH conditions.

### 3.3 Quantitative Phosphate Solubilization

The quantitative determination of soluble phosphorus produced by selected bacterial isolates is presented in Figure 2. The concentration of soluble phosphorus varied among strains and incubation times. In general, higher phosphorus concentrations were observed on day 10 compared with day 5 and day 15. Strain GC3.1 exhibited the highest phosphorus solubilization, reaching approximately 950 mg L^−1^ on day 10. High phosphorus concentrations were also observed in strains GC1.2, GC1.3, and CL4.8, with values ranging from about 800–920 mg L^−1^ on day 10. Moderate phosphorus solubilization was recorded for strains GC1.5, GC2.2, CL2.3, and CL4.3, which produced soluble phosphorus concentrations between approximately 650 and 880 mg L^−1^. In contrast, strains GC4.4 and CL3.1 showed lower phosphorus concentrations, generally below 350 mg L^−1^ across the incubation periods. Overall, the soluble phosphorus concentrations produced by the tested strains ranged from approximately 200 to 950 mg L^−1^ during the observation period, with day 10 showing the highest values for most isolates. Detailed results are available in Table S3.

The quantitative phosphate‐solubilization assay was used as a screening step for strain selection prior to genome sequencing. Bacterial viability and culture purity were not directly assessed during the incubation period, representing a limitation of this assay.

### 3.4 Effect of Salinity on Bacterial Growth and P Solubilization

**FIGURE 3:**
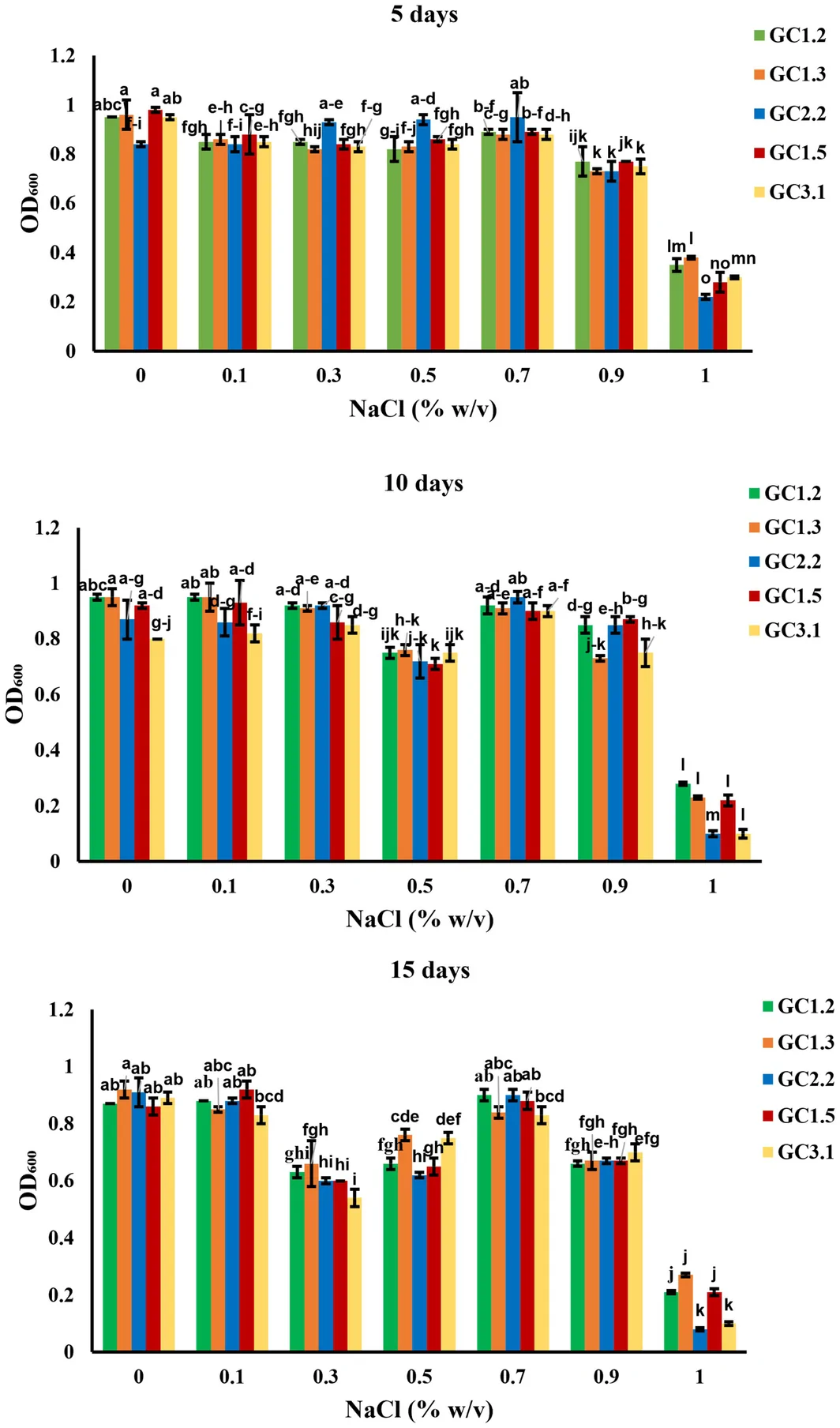
Effect of NaCl concentration on bacterial growth (OD_600_) of selected strains at different incubation times.

Figure 3 shows the effect of different NaCl concentrations on the growth of five phosphate‐solubilizing bacterial strains (GC1.2, GC1.3, GC2.2, GC1.5, and GC3.1), measured by OD_600_ after 5, 10, and 15 days of incubation. Overall, bacterial density generally decreased with increasing NaCl concentration across the incubation periods. In addition, a gradual decline in OD_600_ was also observed over time in the 0% NaCl control, indicating that prolonged incubation may have contributed to the overall reduction in bacterial density. At low salinity levels (0%–0.3% NaCl), the strains maintained relatively high OD_600_ values throughout the incubation period. However, growth was progressively lower at higher NaCl concentrations, particularly from 0.7%–1.0% NaCl, where OD_600_ values were markedly reduced for all strains. Among the tested isolates, GC1.2 and GC1.3 maintained comparatively higher OD_600_ values under moderate salinity levels up to 0.5%–0.7% NaCl.

**FIGURE 4:**
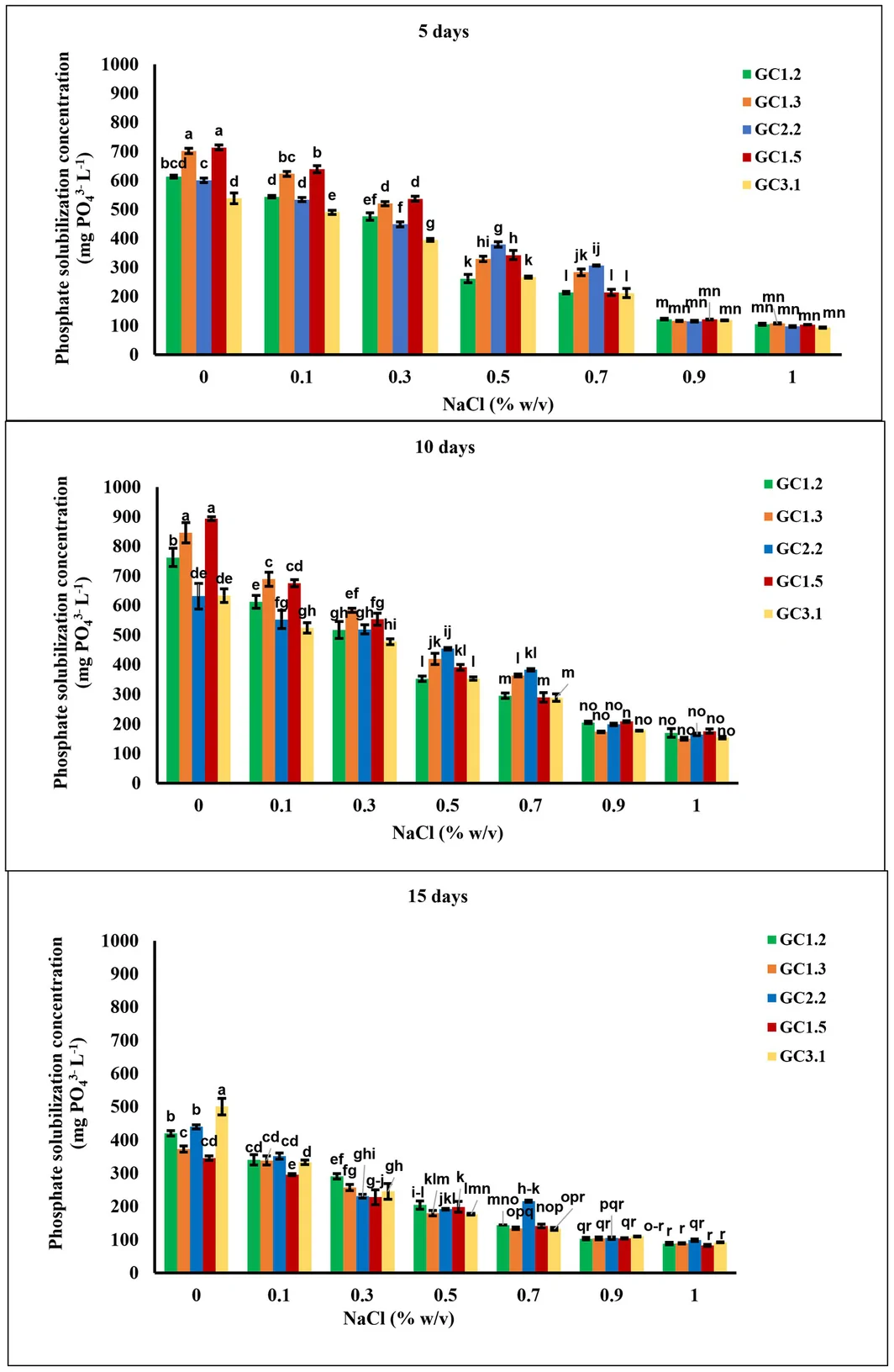
Effect of NaCl concentration on soluble phosphorus production by selected bacterial strains at different incubation times.

Figure 4 presents the effect of NaCl concentration on soluble phosphorus released by the same bacterial strains over 5, 10, and 15 days. Soluble phosphorus concentrations varied among strains and incubation times but generally decreased with increasing NaCl concentration. The highest phosphorus concentrations were observed in media without NaCl, particularly on day 10. As NaCl concentration increased, the amount of soluble phosphorus gradually declined, with the lowest values recorded at 1% NaCl. Overall, the soluble phosphorus concentrations ranged from approximately 100 to over 900 mg L^−1^ depending on strain, incubation time, and salt concentration. The complete dataset is presented in Table S4A–C.

### 3.5 Effect of pH on Bacterial Growth and P Solubilization

**FIGURE 5:**
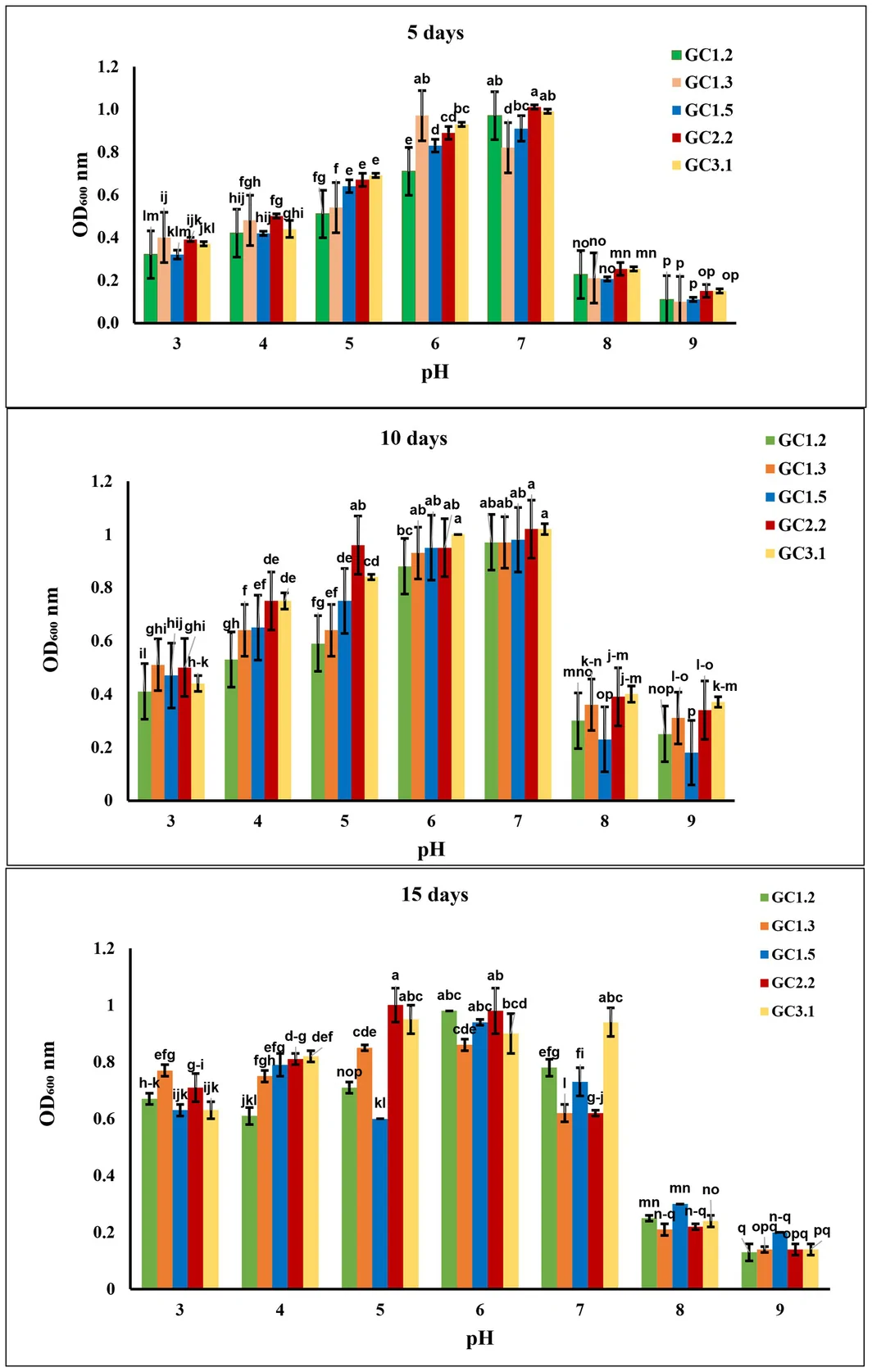
Effect of pH on bacterial growth (OD_600_) of selected strains at different incubation times.

Figure 5 shows that bacterial density varied with medium pH and incubation time. Across all five strains, OD_600_ generally increased from pH 3 to pH 6–7 and then decreased at pH 8–9. On days 5 and 10, most strains reached their highest OD_600_ values at pH 6–7, whereas lower values were observed under strongly acidic and alkaline conditions. By day 15, the same trend remained evident, although strain‐specific differences became more pronounced, with GC1.5 and GC2.2 maintaining relatively higher OD_600_ values within the pH range of 5–7.

**FIGURE 6:**
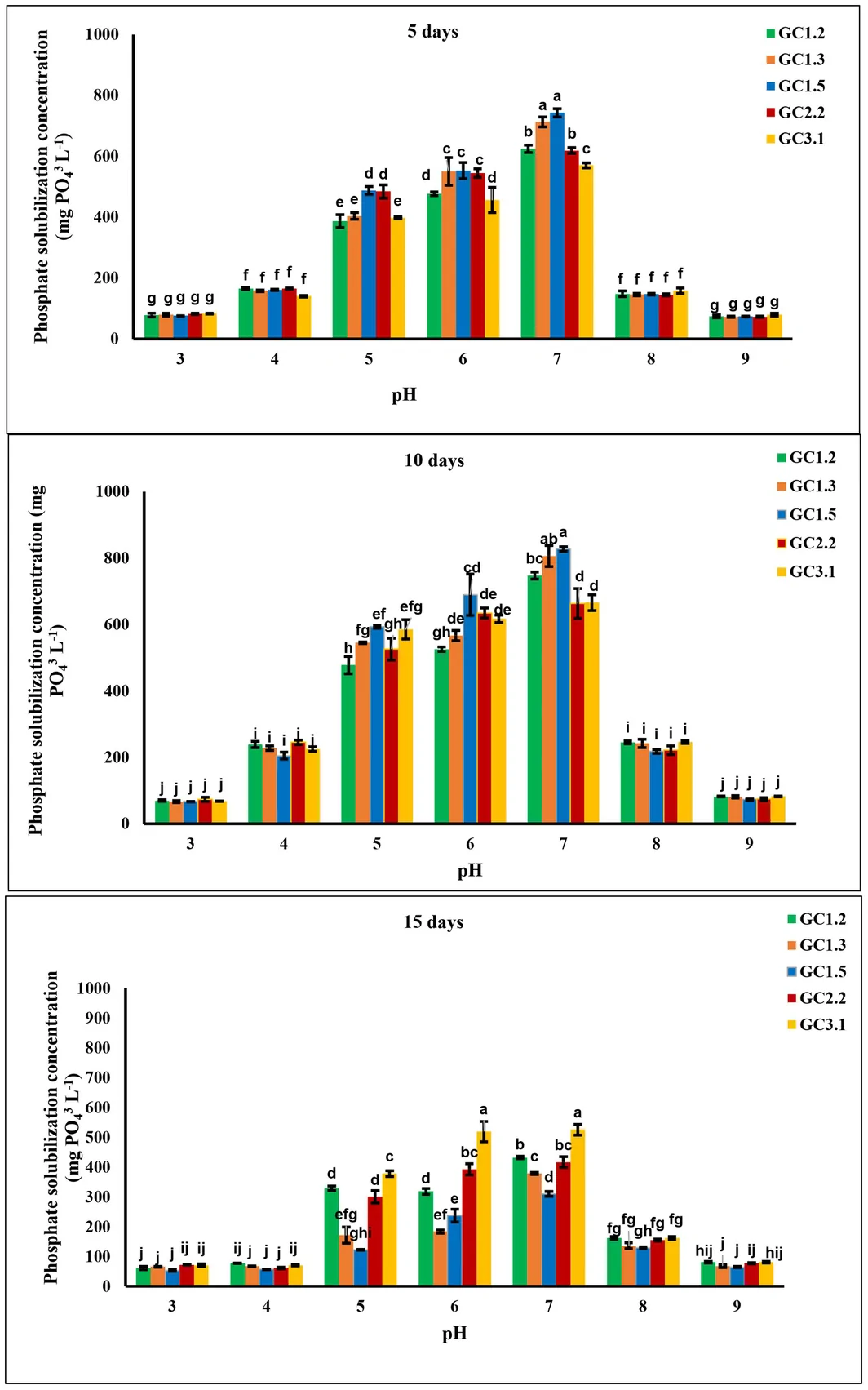
Effect of pH on soluble phosphorus production by selected bacterial strains at different incubation times.

Figure 6 shows that soluble phosphorus concentration was also strongly affected by pH. In general, phosphate solubilization increased from pH 3 to pH 6–7 and declined at pH 8–9 at all sampling times. On day 5, GC1.3 and GC2.2 showed relatively higher soluble phosphorus concentrations at pH 6–7. The highest phosphate‐solubilizing activity was observed on day 10, particularly at pH 7. By day 15, soluble phosphorus concentrations decreased in most treatments, although the overall pattern of maximum solubilization at pH 6–7 remained unchanged. The complete dataset is presented in Table S5A–C.

### 3.6 Molecular Identification of Selected Strains

Strains GC1.2 and GC1.5 were identified as *Pseudomonas* sp., showing sequence identities of 99.89% and 99.79%, respectively. Strain GC1.3 matched *Enterobacter* sp. with 95.93% sequence identity. Strain GC2.2 shared 99.47% sequence identity with *Klebsiella* sp., while strain GC3.1 showed 100% sequence identity with *Acinetobacter* sp. (Table 2).

**TABLE 2:** Summary of the identification results of strains.

Strains	Max score	Query coverage	*E*‐value	Max identities	Sequence length (bp)	GenBank ID	Conclusion
GC1.2	1724	100%	0.0	99.89%	936	PV960527.1	*Pseudomonas* sp.
GC1.3	1733	100%	0.0	95.93%	1538	PX482145.1	*Enterobacter* sp.
GC2.2	1700	99%	0.0	99.47%	938	PV960529.1	*Klebsiella* sp.
GC1.5	1711	100%	0.0	99.79%	933	PV960528.1	*Pseudomonas* sp.
GC3.1	1720	100%	0.0	100%	931	PV960523.1	*Acinetobacter* sp.

Based on the phenotypic characterization and molecular identification results, strain GC1.3 was selected for whole‐genome sequencing. The strain combined high phosphate‐solubilizing activity, broad pH tolerance (3–9), and the ability to maintain phosphate‐solubilizing activity under saline conditions. Moreover, GC1.3 originated from the rhizosphere of wild rice growing in a saline‐affected area of the Mekong Delta. The relatively low 16S rRNA gene sequence identity (95.93%) to the closest reference strain also indicated that whole‐genome sequencing was necessary for accurate taxonomic assignment and comprehensive genomic characterization.

### 3.7 Genome Sequencing and Assembly of Strain GC1.3

#### 3.7.1 Sequencing Data Quality

Short‐read data: Before cleaning, each read direction (R1/R2) yielded 3,706,394 reads, totaling 555,959,100 bp, with read lengths of 35–151 bp, %GC of approximately 55.0–55.2, and %Q30 values of 99.6 (R1) and 94.7 (R2), respectively. After cleaning, 3,596,332 reads remained for each direction, totaling 508,503,696 bp, with %GC of 55.3 and %Q30 remaining high (99.7 for R1; 97.0 for R2), meeting the criteria for de novo assembly (Table 3).

**TABLE 3:** Illumina/MGI sequencing quality metrics of the analyzed sample.

Sample	Total reads	Total bases (bp)	Read length (bp)	%GC	%Q30
Before filtering					
Read R1	3,706,394	555,959,100	35–151	55.2	99.6
Read R2	3,706,394	555,959,100	35–151	55.0	94.7
After filtering					
Read R1	3,596,332	508,503,696	35–151	55.3	99.7
Read R2	3,596,332	508,503,696	35–151	55.3	97.0

Long‐read data (Nanopore): Before cleaning, 37,308 reads were obtained, totaling 278,972,093 bp; after cleaning, 33,778 reads remained with 245,374,740 bp. The median read length increased slightly from 3805.5 bp to 3876.5 bp; the mean read length decreased from 7477.5 bp to 7264.3 bp; and N50 decreased from 16,668 bp to 15,115 bp. The proportion of reads meeting the >Q7 quality threshold after cleaning reached 100% (total ~245.4 Mb), satisfying the threshold for de novo assembly (Table 4).

**TABLE 4:** Nanopore sequencing quality metrics of the analyzed sample.

Long‐read metrics	Before filtering	After filtering
Total reads	37,308.0	33,778.0
Total bases	278,972,093.0	245,374,740.0
Median read length (bp)	3805.5	3876.5
Mean read length (bp)	7477.5	7264.3
Read length standard deviation (stdev) (bp)	10,479.5	9900.6
Maximum read length (bp)	16,668.0	15,115.0
Reads > Q7	37,257 (99.86%), 278.9 Mb	33,778 (100%), 245.4 Mb

#### 3.7.2 Genome Assembly and Annotation

The de novo assembly produced a single circularized contig representing a complete chromosome of 4,778,161 bp, with a GC content of 55.38%. BUSCO analysis identified 435 of 440 complete orthologs (98.9%), including 434 single‐copy BUSCOs (98.6%) and one duplicated BUSCO (0.2%). One BUSCO was fragmented (0.2%), while four were missing (0.9%). CheckM estimated genome completeness at 99.47%, with 0.71% contamination and 0.00% strain heterogeneity. Baseline annotation identified a total of 4375 CDSs, 85 tRNAs, and 25 rRNAs, indicating a genome rich in coding regions and containing the typical complement of functional RNAs found in bacteria (Table 5).

**TABLE 5:** Genome assembly and annotation features of *E. hormaechei* strain GC1.3.

Category	Parameter	Value
Genome assembly	Total genome size	4,778,161 bp
	Average coverage (X)	264.20
	GC content	55.38%
	L50	1
	Number of contigs	1
	Circular contig	1
	BUSCO completeness (%)	98.90
	CheckM completeness (%)	99.47
	CheckM contamination (%)	0.71
	Strain heterogeneity (%)	0.00
Genome annotation	Total genes	4486
	Coding sequences (CDS)	4375
	tRNA	85
	rRNA	25
	tmRNA	1
	Repeat regions	1

**FIGURE 7:**
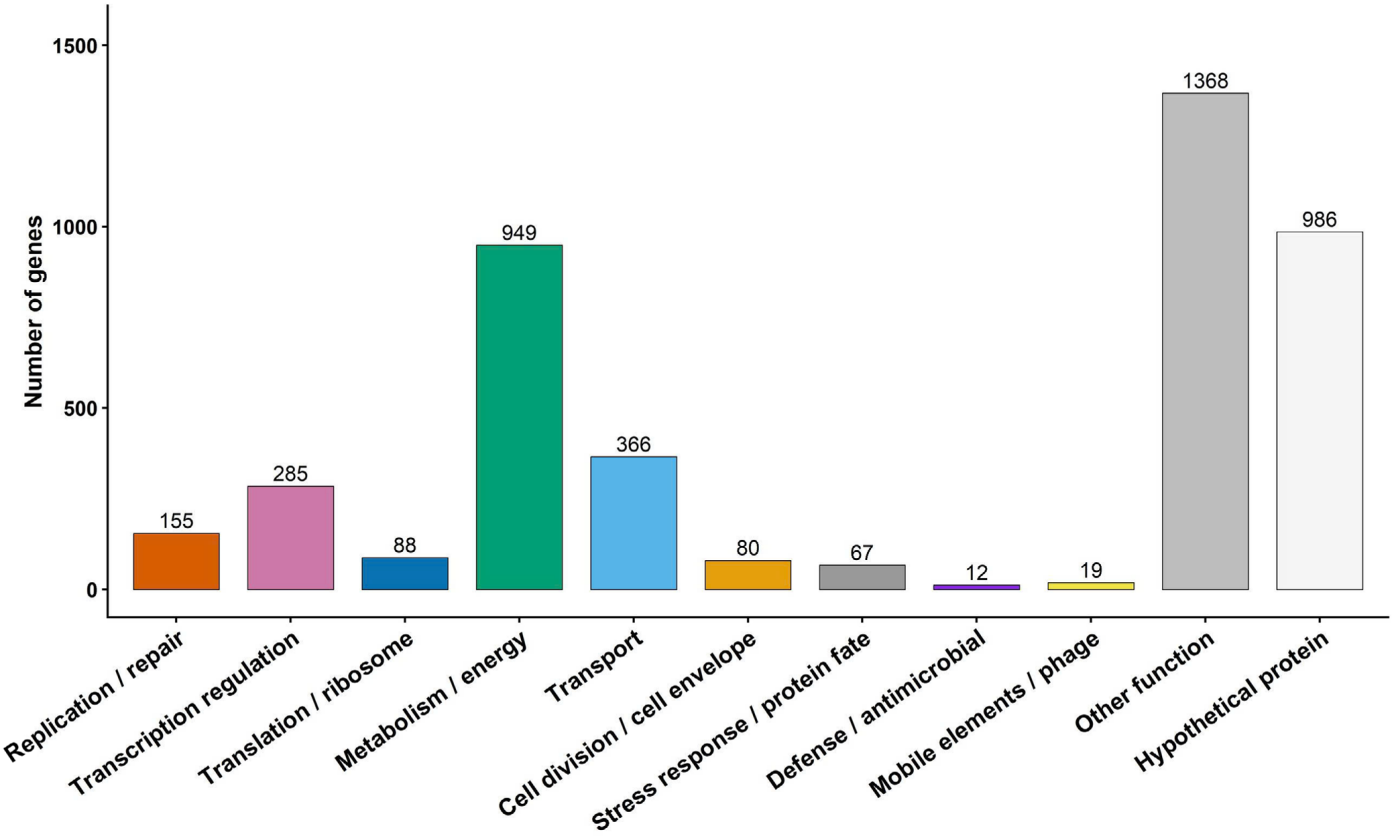
Functional annotation of *E. hormaechei* strain GC1.3, showing the distribution of genes across major biological categories.

Functional annotation classified the predicted genes into multiple functional categories, providing an overall overview of the genomic functional repertoire of strain GC1.3 (Figure 7). The largest proportion of genes was assigned to “Other functions” (1368 genes) and “Hypothetical proteins” (986 genes). Among the annotated functional categories, genes involved in metabolism and energy production (949 genes), transport (366 genes), and transcriptional regulation (285 genes) were the most abundant, whereas relatively few genes were associated with defense, antimicrobial functions, and mobile genetic elements. This global functional distribution provides the genomic context for the subsequent analyses of genes associated with phosphate solubilization, environmental adaptation, and plant growth‐promoting traits.

**FIGURE 8:**
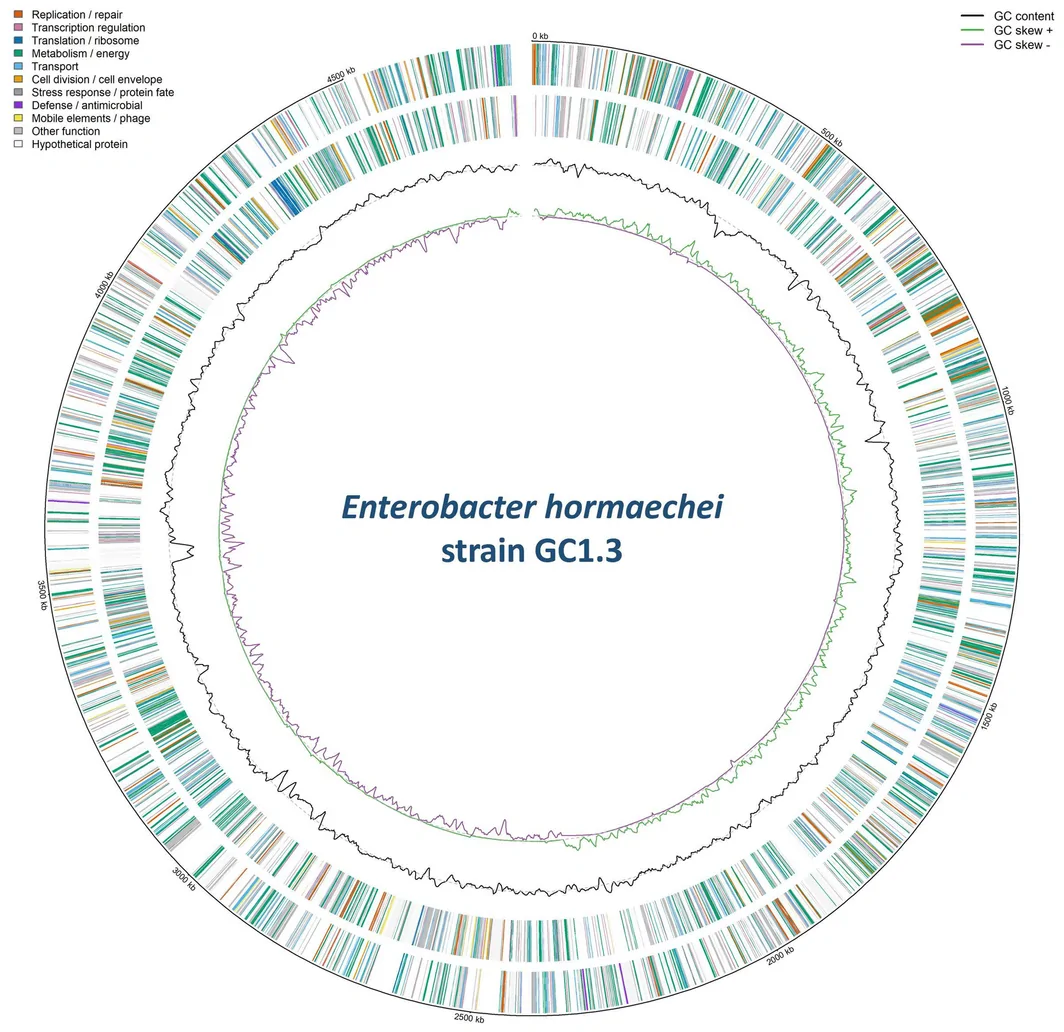
Whole‐genome map of *E. hormaechei* strain GC1.3.

Notably, these functional categories included genes annotated as being involved in phosphate solubilization and phosphate transport, including *gcd*, *pqq*, and *pstSCAB*, whereas a considerable proportion of genes remained annotated as hypothetical or uncharacterized proteins (Figure 8).

#### 3.7.3 Genome‐Based Species Identification

**FIGURE 9:**
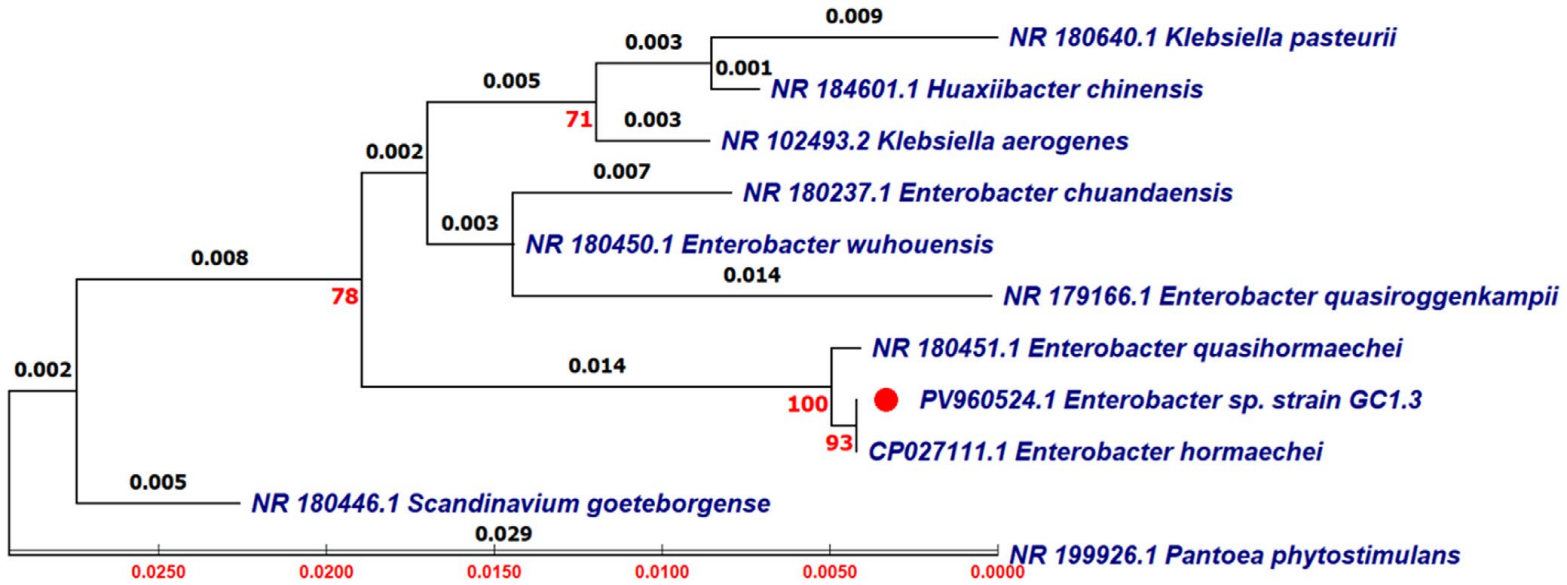
Phylogenetic tree of strain *E. hormaechei* GC1.3.

ANI‐based taxonomic analysis assigned strain GC1.3 to *E. hormaechei* . The genome shared the highest ANI value (98.30%) with *E. hormaechei* KLBMP 4941, whereas the ANI value with the closely related species *E. quasihormaechei* was only 93.64%, below the accepted species‐level threshold. These results support the assignment of strain GC1.3 to *E. hormaechei* (Table 6, Figure 9).

**TABLE 6:** ANI‐based taxonomic identification of strain GC1.3.

Strain	Reference genome	Identity (%)	ANI (%)	Genome coverage (%)	ANI‐based identification	NCBI accession
GC1.3	GCF_001729745.1	95	95.93	90	*E. hormaechei*	JBYFBU000000000
	GCF_900322725.1	95	93.64	86	*E. quasihormaechei*	

### 3.8 Functional Genes Related to Phosphate Metabolism

Genes associated with phosphate solubilization and phosphorus metabolism were identified in the genome of strain GC1.3, including genes involved in inorganic phosphate solubilization, polyphosphate metabolism, and phosphonate transport and utilization (Table 7). The genome contained the *gcd* gene (glucose dehydrogenase; 2391 bp; GC content 57.42%) and *pqqC* (PQQ biosynthesis protein; 756 bp; GC content 59.92%). Genes involved in polyphosphate metabolism, including *ppk* (2061 bp; GC content 53.42%) and *ppx* (1539 bp; GC content 57.70%), were also identified. In addition, a nearly complete *phn* gene cluster, comprising *phnC, phnE, phnF–phnP*, was detected. These genes were annotated as components of the phosphonate transport and carbon–phosphorus lyase pathways.

**TABLE 7:** Genes associated with phosphate solubilization and phosphorus metabolism identified in the genome of *E. hormaechei* strain GC1.3.

Gene	Start position (bp)	End position (bp)	Strand	Predicted function
*gcd*	869,552	871,942	+	Glucose dehydrogenase involved in gluconic acid production and inorganic phosphate solubilization
*pqqC*	2,111,303	2,112,058	−	Pyrroloquinoline quinone biosynthesis protein required for the production of the PQQ cofactor used by glucose dehydrogenase
*ppk*	3,394,715	3,396,775	+	Polyphosphate kinase involved in polyphosphate synthesis and phosphorus storage
*ppx*	3,396,779	3,398,317	+	Exopolyphosphatase involved in polyphosphate degradation and phosphate release
*phnC*	382,809	383,597	−	ATP‐binding component of the phosphonate transport system
*phnE*	380,896	381,675	−	Membrane component of the phosphonate transport system
*phnF*	380,150	380,875	−	Transcriptional regulator of the *phn* operon
*phnG*	379,697	380,149	−	Component of the carbon–phosphorus lyase complex involved in phosphonate utilization
*phnH*	379,116	379,700	−	Component of the carbon–phosphorus lyase complex involved in phosphonate utilization
*phnI*	378,052	379,116	−	Component of the carbon–phosphorus lyase complex involved in phosphonate utilization
*phnJ*	377,214	378,059	−	Carbon–phosphorus lyase catalytic component involved in cleavage of the C–P bond
*phnK*	376,462	377,217	−	ATP‐binding component associated with phosphonate utilization
*phnL*	375,674	376,354	−	Component of the carbon–phosphorus lyase complex involved in phosphonate utilization
*phnM*	374,541	375,677	−	Phosphoribosyl cyclic phosphate phosphodiesterase involved in the carbon–phosphorus lyase pathway
*phnN*	373,993	374,541	−	Ribose‐1,5‐bisphosphate phosphokinase involved in phosphonate metabolism
*phnO*	373,566	374,000	−	Aminoalkylphosphonate N‐acetyltransferase involved in phosphonate metabolism
*phnP*	372,798	373,556	−	Phosphoribosyl 1,2‐cyclic phosphate phosphodiesterase involved in the carbon–phosphorus lyase pathway

In addition to genes associated with phosphate metabolism, several genes involved in environmental stress adaptation were identified in the genome of strain GC1.3, including genes related to compatible‐solute biosynthesis, ion and pH homeostasis, iron regulation, and oxidative stress protection. These genes and their predicted functions are presented in Table S6.

Comparative genomic analysis showed differences in the numbers of annotated functional genes among the three *E. hormaechei* genomes (Table 8). Strain GC1.3 contained 411 annotated genes related to phosphate metabolism, compared with 395 genes in *E. hormaechei* DSM 14563 and 309 genes in *E. hormaechei* FDAARGOS 1433, including genes such as *gcd, pqq, phoPR*, and *pstSCAB*. The numbers of annotated stress‐related genes were 67 in GC1.3, 61 in DSM 14563, and 45 in FDAARGOS 1433. Similarly, 260 genes associated with transport and environmental adaptation were identified in GC1.3, compared with 238 and 216 genes in DSM 14563 and FDAARGOS 1433, respectively. Plant growth‐promoting (PGP) genes were represented by four annotated genes in both GC1.3 and DSM 14563, and two genes in FDAARGOS 1433.

**TABLE 8:** Comparative summary of key functional genes including core phosphate genes.

Category	Representative genes (including key genes)	*E. hormaechei* strain GC1.3	*E. hormaechei* strain DSM 14563	*E. hormaechei* strain FDAARGOS 1433
Phosphate metabolism (P)	*phoU*, *pstA*, *pstB*, *pstC*, *pstS*, *phnC*	411	395	309
Plant growth promotion (PGP)	*viaA*, *dacD*, *diaA*	4	4	2
Stress tolerance	*gmuB*, *ebgA*, *ebgC*, *wbjC*, *atpD*, *bglK*	67	61	45
Transport & adaptation	*araQ*, *bepE*, *shlB*, *sugB*, *oqxB*, *bicA*	260	238	216

### 3.9 KEGG Pathway Reconstruction

KEGG pathway reconstruction identified genes assigned to the ABC transporter (ko02010) and two‐component regulatory system (ko02020) pathways in the genome of *E. hormaechei* GC1.3. Genes associated with inorganic phosphate and phosphonate transport were identified within the ABC transporter pathway (Figure S1). Genes assigned to two‐component regulatory systems related to phosphate limitation, osmotic stress, nitrogen metabolism, and carbon utilization were also identified (Figure S2).

## 4 Discussion

The outstanding phosphate‐solubilizing performance of strain GC1.3 reflects not merely a high metabolic rate but a genome‐level optimization of phosphorus cycling functions, distinguishing it from typical PSB. Rather than relying on a single biochemical pathway, GC1.3 appears to integrate multiple functional modules into a coordinated system that maximizes phosphorus mobilization, acquisition, and utilization under variable environmental conditions.

At the core of this system is the PQQ‐dependent glucose oxidation pathway, driven by the functional coupling of the *gcd* gene and the *pqq* gene cluster. The *gcd*‐encoded glucose dehydrogenase catalyzes the oxidation of glucose into gluconic acid, while the *pqq* cluster, particularly *pqqC*, controls the biosynthesis of the essential cofactor PQQ. Recent genomic studies have demonstrated that *pqqC* is a key determinant of phosphate‐solubilizing capacity, as it directly regulates the formation of 2‐keto‐D‐gluconic acid, the most effective organic acid for mineral phosphate dissolution (Chen et al. 2024). In parallel, metagenomic evidence has identified *gcd* as one of the dominant functional genes governing phosphorus bioavailability in soil ecosystems, strongly linked to increases in soluble phosphorus pools (Liang et al. 2020). These findings collectively indicate that the *gcd*–*pqq* system functions as the central biochemical engine of phosphate solubilization, where gene co‐occurrence and coordinated expression are more critical than the presence of either gene alone.

Beyond this central pathway, GC1.3 exhibits a multi‐dimensional phosphorus acquisition strategy, supported by the presence of additional gene systems identified in Table 5. The detection of phosphonate transport genes (*phnC* and *phnD*) expands the substrate spectrum of the strain, allowing access to alternative phosphorus sources beyond inorganic phosphate. Simultaneously, the presence of polyphosphate metabolism genes (*ppk* and *ppx*) enables intracellular phosphorus storage and controlled remobilization after phosphate uptake. Together, these functional traits integrate phosphorus solubilization, uptake, storage, and reutilization, thereby enhancing phosphorus‐use efficiency and contributing to phosphorus cycling in the rhizosphere rather than constituting a closed‐loop phosphorus cycling system. Such integration of phosphorus acquisition and intracellular phosphorus management has been increasingly recognized as a hallmark of highly efficient rhizosphere bacteria (Pan and Cai 2023).

A defining feature of GC1.3 is the tight coupling between phosphorus metabolism and stress adaptation mechanisms. The genome encodes multiple systems involved in osmotic balance and environmental resilience, including glycine betaine synthesis (*betA*, *betB*), trehalose biosynthesis (*otsA*, *otsB*), and ion transport systems (*kdpA/kdpB*). These genes play a critical role in maintaining intracellular stability under salinity and osmotic stress, thereby preserving the functionality of the *gcd*–*pqq* pathway. Under such conditions, phosphate solubilization is often limited by reduced metabolic flux and enzyme instability (Wang et al. 2025), yet strains capable of maintaining osmotic homeostasis can sustain organic acid production and proton extrusion. This explains why efficient PSB are often characterized not only by metabolic capability but also by their ability to decouple functional performance from environmental stress constraints (Zhao et al. 2025).

In addition, the presence of metal homeostasis and oxidative stress genes (*fur*, *mntH*) further enhances metabolic robustness by regulating intracellular redox balance and metal ion availability. These factors are particularly important in phosphate solubilization, as metal ions such as Fe^3+^ and Al^3+^ directly influence phosphorus binding and release dynamics. The ability to regulate these ions at the cellular level provides an additional layer of control over phosphate availability, linking intracellular homeostasis with extracellular nutrient cycling.

From a regulatory perspective, GC1.3 also demonstrates a high degree of environmental responsiveness. The identification of ABC transporters and two‐component regulatory systems, including the PhoR/PhoB regulon, suggests that this strain can dynamically adjust gene expression in response to phosphate limitation and environmental stress (Stock et al. 2000). This regulatory flexibility enables the coordinated activation of solubilization, transport, and stress‐response pathways, ensuring efficient phosphorus acquisition even under fluctuating conditions. Such genome‐scale coordination is increasingly viewed as a key evolutionary strategy in rhizosphere bacteria, where nutrient availability and environmental factors are highly variable (Chen et al. 2024).

Comparative genomic analysis further underscores the ecological and functional distinctiveness of GC1.3. Although phylogenetically affiliated with *E. hormaechei* within the *E. cloacae* *complex* (ECC), this strain exhibits a functionally enriched genome architecture, characterized by the integration of genes involved in phosphorus metabolism, environmental stress adaptation, and regulatory control. Previous studies have demonstrated that members of the ECC can share high genomic similarity (e.g., based on ANI) while exhibiting substantial differences in accessory gene content, which drive ecological diversification and niche‐specific adaptation (Paauw et al. 2008). In particular, comparative genome analyses have shown that while core genes are conserved, genes related to virulence, environmental adaptation, and metabolic specialization are often variably distributed across strains, reflecting adaptation to different ecological niches (Mustafa et al. 2020).

In this context, the co‐occurrence of phosphorus‐cycling genes (*gcd*, *pqqC*, *phn*, *ppk/ppx*) together with stress‐response modules in GC1.3 suggests a genome‐level functional integration, enabling tight coupling between nutrient acquisition and environmental resilience. Such integrative genomic configurations have been widely recognized as adaptive strategies in rhizosphere‐associated bacteria, where metabolic flexibility and stress tolerance are essential for maintaining functional stability under fluctuating environmental conditions (Paauw et al. 2008). Taken together, these features indicate that GC1.3 represents a specialized ecological strategist, whose genome is optimized for efficient phosphorus cycling and sustained functionality in stress‐prone environments.

Overall, the superior functional capacity of GC1.3 can be attributed to a synergistic and hierarchical genomic network, where the central *gcd‐pqq* pathway is supported by complementary systems for phosphorus acquisition, storage, stress tolerance, and regulatory control. This integration supports the concept that phosphate solubilization is not a single‐gene trait but an emergent property of coordinated metabolic and regulatory interactions at the genome scale. Such a configuration provides a mechanistic explanation for the exceptional efficiency of GC1.3 and highlights its potential as a robust biofertilizer candidate for sustainable agriculture in nutrient‐limited and environmentally stressed soils (Qiu et al. 2024).

## 5 Conclusions

This study demonstrates that the exceptional phosphate‐solubilizing capacity of strain GC1.3 is not attributable to a single metabolic trait, but rather to a genome‐scale integration of complementary functional systems. The coordinated interaction between the central *gcd‐pqq* driven organic acid pathway and auxiliary modules for phosphorus acquisition (*phn*, *pst*), intracellular storage (*ppk/ppx*), and environmental stress adaptation provides a robust mechanistic basis for its superior performance. In particular, the functional coupling of *gcd* and *pqqC* highlights a key biochemical axis governing gluconic acid‐mediated phosphate solubilization, while regulatory and stress‐response networks suggest a genetic capacity for responding to environmental stress, although this potential requires further phenotypic validation. These findings support the emerging concept that phosphate solubilization is an emergent property of integrated metabolic and regulatory networks, rather than a single‐gene function. Consequently, GC1.3 represents a genome‐optimized ecological strategist for phosphorus cycling, with strong potential for application as a promising biofertilizer candidate whose performance under environmental stress warrants further investigation.

## Author Contributions

**Xuyen Thi Hong Nguyen:** data curation (equal), formal analysis (equal), investigation (equal), methodology (equal), writing – original draft (equal), writing – review and editing (equal). **Tan Khang Do:** funding acquisition (equal), methodology (equal), supervision (equal), writing – review and editing (equal). **Giang Thi Tran:** methodology (equal), project administration (equal), supervision (equal), writing – review and editing (equal).

## Funding

This study was supported by the project “Collecting, conserving and developing microbial genetic resources in wild rice populations in Can Tho City” from Can Tho Science and Technology Department (DP2025‐04).

## Conflicts of Interest

The authors declare no conflicts of interest.

## Supporting information


**Figure S1:** Functional map of ABC transporters (ko02010) in the genome of strain *E. hormaechei* .
**Figure S2:** Functional map of the Two‐component system (ko02020) in the genome of strain *E. hormaechei* .
Green boxes represent KEGG Orthologs (KOs) annotated based on KofamScan results, indicating the presence of transport systems associated with phosphate and phosphonate.
**Table S1:** Geographic coordinates and sampling locations of wild rice ( *Oryza rufipogon* ) rhizosphere samples collected in Tien Giang Province, Vietnam.
**Table S2:** Phosphate solubilization index (PSI) of bacterial isolates at different incubation times.
**Table S3:** Quantitative analysis of soluble phosphorus produced by phosphate‐solubilizing bacterial strains isolated from the rhizosphere soil of wild rice in Vietnam.
**Table S4A:** Effects of NaCl concentration on growth (OD_600_) and soluble phosphorus production of phosphate‐solubilizing bacteria isolated from wild rice rhizosphere (5 days).
**Table S4B:** Effects of NaCl concentration on growth (OD_600_) and soluble phosphorus production of phosphate‐solubilizing bacteria isolated from wild rice rhizosphere (10 days).
**Table S4C:** Effects of NaCl concentration on growth (OD_600_) and soluble phosphorus production of phosphate‐solubilizing bacteria isolated from wild rice rhizosphere (15 days).
**Table S5A:** Effects of pH on growth (OD_600_) and soluble phosphorus production of phosphate‐solubilizing bacteria isolated from wild rice rhizosphere (5 days).
**Table S5B:** Effects of pH on growth (OD_600_) and soluble phosphorus production of phosphate‐solubilizing bacteria isolated from wild rice rhizosphere (10 days).
**Table S5C:** Effects of pH on growth (OD_600_) and soluble phosphorus production of phosphate‐solubilizing bacteria isolated from wild rice rhizosphere (15 days).
**Table S6:** Genes associated with environmental stress adaptation identified in the genome of *Enterobacter hormaechei* strain GC1.3.

## Data Availability

The complete genome sequence of *E. hormaechei* strain GC1.3 has been deposited in the NCBI GenBank database under accession number JBYFBU000000000. The associated BioProject and BioSample accession numbers are PRJNA1465318 and SAMN59741156, respectively. Additional datasets supporting the findings of this study are available in the Figshare repository at https://doi.org/10.6084/m9.figshare.31996104.
