# Supplementary material for: Genome‐Resolved Insights Into Phosphate‐Solubilizing Bacteria From Wild Rice Rhizosphere Reveal Adaptive Strategies for Phosphorus Cycling Under Acid‐Saline Stress in the Mekong Delta, Vietnam
Source: Ecol Evol. 2026 Sep 23;16(9):e74327. doi: 10.1002/ece3.74327 (PMC13601653; doi:10.1002/ece3.74327)
Supplement: Supplementary file 1 [file ECE3-16-e74327-s001.docx]

**Table S1.** Geographic coordinates and sampling locations of wild rice (*Oryza rufipogon*) rhizosphere samples collected in Tien Giang Province, Vietnam.

| **Sample ID** | **No. of plants sampled** | **Latitude (°N)** | **Longitude (°E)** | **Sampling location** | **Administrative unit** |
| --- | --- | --- | --- | --- | --- |
| GC1 | 6 | 10.315139 | 106.734944 | Tan Hoa | Tan Hoa Commune, Go Cong Dong District, Tien Giang Province, Vietnam |
| GC2 | 4 | 10.284806 | 106.708250 | Phuoc Trung | Phuoc Trung Commune, Go Cong Dong District, Tien Giang Province, Vietnam |
| GC3 | 4 | 10.293889 | 106.695889 | Phuoc Trung | Phuoc Trung Commune, Go Cong Dong District, Tien Giang Province, Vietnam |
| GC4 | 5 | 10.276417 | 106.695444 | Phu Dong | Phu Dong Commune, Go Cong Dong District, Tien Giang Province, Vietnam |
| CL1 | 5 | 10.396750 | 106.078989 | Hiep Duc | Hiep Duc Commune, Cai Lay District, Tien Giang Province, Vietnam |
| CL2 | 6 | 10.393694 | 106.078111 | Hiep Duc | Hiep Duc Commune, Cai Lay District, Tien Giang Province, Vietnam |
| CL3 | 5 | 10.038167 | 106.112194 | Hiep Duc | Hiep Duc Commune, Cai Lay District, Tien Giang Province, Vietnam |
| CL4 | 8 | 10.396750 | 106.078990 | Hiep Duc | Hiep Duc Commune, Cai Lay District, Tien Giang Province, Vietnam |

*Note: Geographic coordinates and administrative information for wild rice (Oryza rufipogon) rhizosphere sampling sites in Tien Giang Province, Vietnam. Each sampling location was considered one biologically independent composite sample.*

**Table S2.** Phosphate solubilization index (PSI) of bacterial isolates at different incubation times.

| **No.** | **Strain** | **5 days** | **10 days** | **15 days** |
| --- | --- | --- | --- | --- |
| 1 | GC1.1 | 1.18^i-l^ ± 0.11 | 1.29^h-k^ ± 0.06 | 1.26^h-k^ ± 0.11 |
| 2 | GC1.2 | 2.05^b^ ± 0.08 | 2.49^bc^ ± 0.15 | 2.38^b^ ± 0.16 |
| 3 | GC1.3 | 1.61^c-f^ ± 0.15 | 1.88^def^ ± 0.13 | 1.59^e-j^ ± 0.16 |
| 4 | GC1.4 | 1.52^c-h^ ± 0.09 | 1.63^e-i^ ± 0.15 | 1.46^f-k^ ± 0.13 |
| 5 | GC1.5 | 1.73^c^ ± 0.07 | 2.12^cd^ ± 0.10 | 2.08^bc^ ± 0.13 |
| 6 | GC1.6 | 1.47^c-i^ ± 0.10 | 1.43^h-k^ ± 0.08 | 1.41^g-k^ ± 0.04 |
| 7 | GC2.1 | 1.07^klm^ ± 0.01 | 1.14^h-k^ ± 0.01 | 1.24^jk^ ± 0.06 |
| 8 | GC2.2 | 1.63^c-f^ ± 0.12 | 1.94^de^ ± 0.19 | 1.85^cde^ ± 0.14 |
| 9 | GC2.3 | 1.62^c-f^ ± 0.14 | 1.60^e-i^ ± 0.09 | 1.57^e-j^ ± 0.07 |
| 10 | GC2.4 | 1.13^j-m^ ± 0.00 | 1.26^h-k^ ± 0.04 | 1.12^kl^ ± 0.06 |
| 11 | GC3.1 | 1.65^cde^ ± 0.10 | 1.93^de^ ± 0.12 | 1.66^d-g^ ± 0.12 |
| 12 | GC3.2 | 1.56^c-g^ ± 0.01 | 1.65^e-h^ ± 0.10 | 1.63^d-h^ ± 0.06 |
| 13 | GC3.3 | 1.00^m^ ± 0.02 | 1.06^h-k^ ± 0.00 | 1.20^l^ ± 0.07 |
| 14 | GC3.4 | 1.55^c-h^ ± 0.14 | 1.63^e-i^ ± 0.07 | 1.47^f-k^ ± 0.12 |
| 15 | GC4.1 | 1.37^e-k^ ± 0.11 | 1.19^h-k^ ± 0.11 | 1.14^kl^ ± 0.16 |
| 16 | GC4.2 | 1.29^g-l^ ± 0.07 | 1.31^h-k^ ± 0.12 | 1.13^kl^ ± 0.15 |
| 17 | GC4.3 | 1.20^i-l^ ± 0.11 | 1.18^h-k^ ± 0.09 | 1.24^jk^ ± 0.13 |
| 18 | GC4.4 | 1.46^c-i^ ± 0.03 | 1.85^d-g^ ± 0.16 | 1.37^g-k^ ± 0.14 |
| 19 | GC4.5 | 1.61^c-f^ ± 0.04 | 1.54^f-j^ ± 0.13 | 1.61^d-i^ ± 0.14 |
| 20 | CL1.1 | 1.26^g-l^ ± 0.10 | 1.33^h-k^ ± 0.09 | 1.35^g-k^ ± 0.12 |
| 21 | CL1.3 | 1.25^h-l^ ± 0.07 | 1.34^h-k^ ± 0.06 | 1.25^ijk^ ± 0.02 |
| 22 | CL1.4 | 1.09^klm^ ± 0.00 | 1.35^h-k^ ± 0.02 | 1.31^g-k^ ± 0.02 |
| 23 | CL1.5 | 1.27^g-l^ ± 0.05 | 1.36^h-k^ ± 0.07 | 1.27^h-k^ ±0.07 |
| 24 | CL1.6 | 1.27^g-l^ ± 0.05 | 1.36^h-k^ ± 0.03 | 1.29^h-k^ ± 0.07 |
| 25 | CL2.1 | 1.10^klm^ ± 0.10 | 1.27^h-k^ ± 0.06 | 1.11^kl^ ± 0.08 |
| 26 | CL2.2 | 1.27^g-l^ ± 0.07 | 1.39^h-k^ ± 0.09 | 1.42^g-k^ ± 0.09 |
| 27 | CL2.3 | 1.72^cd^ ± 0.15 | 2.19^bcd^ ± 0.18 | 1.96^cd^ ± 0.14 |
| 28 | CL2.4 | 1.00^lm^ ± 0.08 | 1.33^h-k^ ± 0.12 | 1.31^g-k^ ± 0.08 |
| 29 | CL2.5 | 1.15^jkl^ ± 0.10 | 1.35^h-k^ ± 0.07 | 1.35^g-k^ ± 0.12 |
| 30 | CL2.6 | 1.42^d-j^ ± 0.11 | 1.61^e-i^ ± 0.15 | 1.57^e-j^ ± 0.12 |
| 31 | CL3.1 | 2.50^a^ ± 0.06 | 3.00^a^ ± 0.10 | 2.91^a^ ± 0.09 |
| 32 | CL3.2 | 1.28^g-l^ ± 0.05 | 1.32^h-k^ ± 0.01 | 1.45^f-k^ ± 0.06 |
| 33 | CL3.3 | 1.36^e-k^ ± 0.07 | 1.36^h-k^ ± 0.14 | 1.42^g-k^ ± 0.13 |
| 34 | CL3.4 | 1.36^e-k^ ± 0.06 | 1.41^h-k^ ± 0.13 | 1.38^g-k^ ± 0.05 |
| 35 | CL3.5 | 1.34^f-k^ ± 0.10 | 1.42^h-k^ ± 0.12 | 1.39^g-k^ ± 0.05 |
| 36 | CL4.2 | 1.25^h-l^ ± 0.09 | 1.40^h-k^ ± 0.11 | 1.34^g-k^ ± 0.03 |
| 37 | CL4.3 | 1.48^c-i^ ± 0.09 | 1.89^def^ ± 0.11 | 1.80^c-f^ ± 0.17 |
| 38 | CL4.4 | 1.33^f-k^ ± 0.03 | 1.44^h-k^ ± 0.12 | 1.32^g-k^ ± 0.09 |
| 39 | CL4.5 | 1.28^g-l^ ± 0.08 | 1.38^h-k^ ± 0.09 | 1.30^e-j^ ± 0.15 |
| 40 | CL4.6 | 1.34^f-k^± 0.12 | 1.49^g-k^ ± 0.13 | 1.36^g-k^ ± 0.08 |
| 41 | CL4.7 | 1.40^klm^ ± 0.06 | 1.40^h-k^ ± 0.09 | 1.38^g-k^ ± 0.08 |
| 42 | CL4.8 | 2.11^b^ ± 0.19 | 2.54^b^ ± 0.18 | 2.39^b^ ± 0.10 |
| 43 | CL4.9 | 1.18^i-l^ ± 0.01 | 1.37^h-k^ ± 0.06 | 1.22^jk^ ± 0.07 |

***Note:*** *Values are presented as mean ± standard deviation (SD). Different superscript letters within the same column indicate significant differences among bacterial strains (P < 0.05).*

**Table S3.** Quantitative analysis of soluble phosphorus produced by phosphate-solubilizing bacterial strains isolated from the rhizosphere soil of wild rice in Viet Nam.

| **No.** | **Strain** | **Soluble phosphorus (mg L⁻¹)** | | |
| --- | --- | --- | --- | --- |
|  |  | **Day 5** | **Day 10** | **Day 15** |
| 1 | GC1.2 | 852.90^a^ ± 8.40 | 919.12^a^ ± 50.8 | 418.05^de^ ± 13.00 |
| 2 | GC1.3 | 768.73^b^ ± 9.80 | 809.91^a^ ± 14.2 | 315.39^f^ ± 7.70 |
| 3 | GC1.5 | 650.84^c^ ±1 0.1 | 897.52^a^ ±14.16 | 430.26^d^ ± 28.00 |
| 4 | GC2.2 | 751.77^b^ ± 10.5 | 813.00^a^ ± 37.9 | 370.85^e^ ± 16.8 |
| 5 | GC3.1 | 744.99^b^ ± 45.4 | 954.16^a^ ± 18.4 | 445.15^d^ ± 41.9 |
| 6 | GC4.4 | 264.96^g^ ± 24.8 | 298.11^c^ ± 18.6 | 221.02^g^ ± 21.4 |
| 7 | CL2.3 | 496.87^e^ ± 2.80 | 669.06^ab^ ± 1.70 | 578.21^bc^ ± 6.80 |
| 8 | CL3.1 | 588.43^d^ ± 3.20 | 668.11^bc^ ± 6.20 | 659.46^a^ ± 2.60 |
| 9 | CL4.3 | 427.19^f^ ± 1.30 | 715.21^ab^ ± 3.20 | 554.49^c^ ± 6.80 |
| 10 | CL4.8 | 521.80^e^ ± 4.30 | 803.64^a^ ± 2.52 | 614.89^ab^ ± 1.30 |

***Note:*** *Values are presented as mean ± standard deviation (SD). Different superscript letters within the same column indicate significant differences among bacterial strains (P < 0.05).*

**Table S4A.** Effects of NaCl concentration on growth (OD₆₀₀) and soluble phosphorus production of phosphate-solubilizing bacteria isolated from wild rice rhizosphere (5 days).

| **No.** | **Strain** | **NaCl concentration (%)** | **Evaluation parameters** | |
| --- | --- | --- | --- | --- |
|  |  |  | **OD₆₀₀** | **Soluble phosphorus (mg L⁻¹)** |
| 1 | GC1.2 | 0 | 0.95^abc^±0.00 | 613.56^bcd^±5.41 |
| 2 |  | 0.1 | 0.85^fgh^±0.03 | 543.95^d^±4.09 |
| 3 |  | 0.3 | 0.85^fgh^±0.01 | 475.96^ef^±12.90 |
| 4 |  | 0.5 | 0.82^g-j^±0.05 | 261.35^k^±14.31 |
| 5 |  | 0.7 | 0.89^b-f^±0.01 | 213.71^l^±4.60 |
| 6 |  | 0.9 | 0.77^ijk^±0.06 | 121.83^m^±2.70 |
| 7 |  | 1 | 0.35^lm^±0.01 | 104.72^mn^±3.68 |
| 8 | GC1.3 | 0 | 0.96^a^±0.06 | 702.21^a^±9.20 |
| 9 |  | 0.1 | 0.86^e-h^±0.02 | 622.56^bc^±9.20 |
| 10 |  | 0.3 | 0.82^hij^±0.01 | 520.35^d^±7.08 |
| 11 |  | 0.5 | 0.83^f-j^±0.02 | 329.64^hi^±8.85 |
| 12 |  | 0.7 | 0.88^c-g^±0.02 | 283.77^jk^±11.38 |
| 13 |  | 0.9 | 0.73^k^±0.01 | 115.40^mn^±2.04 |
| 14 |  | 1 | 0.38^l^±0.01 | 107.08^mn^±1.77 |
| 15 | GC1.5 | 0 | 0.98^a^±0.01 | 713.63^a^±8.39 |
| 16 |  | 0.1 | 0.88^c-g^±0.08 | 638.49^b^±12.17 |
| 17 |  | 0.3 | 0.84^fgh^±0.02 | 536.87^d^±8.17 |
| 18 |  | 0.5 | 0.86^fgh^±0.01 | 342.92^h^±15.95 |
| 19 |  | 0.7 | 0.89^b-f^±0.01 | 214.60^l^±10.73 |
| 20 |  | 0.9 | 0.77^jk^±0.00 | 121.24^mn^±1.77 |
| 21 |  | 1 | 0.28^no^±0.00 | 102.95^mn^±1.02 |
| 22 | GC2.2 | 0 | 0.84^f-i^±0.01 | 600.53^c^±8.08 |
| 23 |  | 0.1 | 0.84^f-i^±0.03 | 533.80^d^±7.19 |
| 24 |  | 0.3 | 0.93^a-e^±0.01 | 448.67^f^±9.20 |
| 25 |  | 0.5 | 0.94^a-d^±0.02 | 379.80^g^±9.21 |
| 26 |  | 0.7 | 0.95^ab^±0.10 | 307.37^ij^±2.04 |
| 27 |  | 0.9 | 0.73^k^±0.00 | 114.75^mn^±2.70 |
| 28 |  | 1 | 0.22^o^±0.01 | 97.05^mn^±2.70 |
| 29 | GC3.1 | 0 | 0.95^ab^±0.01 | 538.73^d^±18.64 |
| 30 |  | 0.1 | 0.87^e-h^±0.02 | 490.41^e^±7.15 |
| 31 |  | 0.3 | 0.83^f-j^±0.02 | 395.57^g^±5.31 |
| 32 |  | 0.5 | 0.84^fgh^±0.02 | 267.26^k^±3.54 |
| 33 |  | 0.7 | 0.88^d-h^±0.02 | 211.95^l^±15.56 |
| 34 |  | 0.9 | 0.75^k^±0.03 | 118.29^mn^±1.02 |
| 35 |  | 1 | 0.30^mn^±0.00 | 93.51^n^±2.04 |

***Note:*** *Values are presented as mean ± standard deviation (SD). Different superscript letters within the same column indicate significant differences among bacterial strains (P < 0.05).*

**Table S4B.** Effects of NaCl concentration on growth (OD₆₀₀) and soluble phosphorus production of phosphate-solubilizing bacteria isolated from wild rice rhizosphere (10 days).

| **No.** | **Strain** | **NaCl concentration (%)** | | | **Evaluation parameters** | | |
| --- | --- | --- | --- | --- | --- | --- | --- |
|  |  |  |  |  | **OD₆₀₀** | **Soluble phosphorus (mg L⁻¹)** | |
| 1 | GC1.2 | | 0 | 0.95^abc^±0.01 | | | 762.53^b^±31.08 |
| 2 |  |  | 0.1 | 0.95^ab^±0.01 | | | 612.39^e^±21.24 |
| 3 |  |  | 0.3 | 0.92^a-d^±0.01 | | | 517.25^gh^±28.39 |
| 4 |  |  | 0.5 | 0.75^ijk^±0.02 | | | 353.09^l^±9.20 |
| 5 |  |  | 0.7 | 0.92^a-d^±0.03 | | | 295.57^m^±9.37 |
| 6 |  |  | 0.9 | 0.85^d-g^±0.03 | | | 204.86^no^±1.53 |
| 7 |  |  | 1 | 0.28^l^±0.00 | | | 169.46^no^±14.62 |
| 8 | GC1.3 | | 0 | 0.95^a^±0.03 | | | 845.60^a^±34.14 |
| 9 |  |  | 0.1 | 0.95^ab^±0.05 | | | 689.20^c^±23.90 |
| 10 |  |  | 0.3 | 0.91^a-e^±0.01 | | | 584.07^ef^±7.08 |
| 11 |  |  | 0.5 | 0.76^h-k^±0.02 | | | 420.20^jk^±18.93 |
| 12 |  |  | 0.7 | 0.91^a-e^±0.02 | | | 363.57^l^±5.11 |
| 13 |  |  | 0.9 | 0.73^jk^±0.01 | | | 173.00^no^±3.07 |
| 14 |  |  | 1 | 0.23^l^±0.00 | | | 150.00^o^±5.31 |
| 15 | GC1.5 | | 0 | 0.92^a-d^±0.01 | | | 893.80^a^±6.13 |
| 16 |  |  | 0.1 | 0.93^a-d^±0.08 | | | 675.66^cd^±12.17 |
| 17 |  |  | 0.3 | 0.86^c-g^±0.06 | | | 553.39^fg^±20.44 |
| 18 |  |  | 0.5 | 0.71^k^±0.02 | | | 391.30^kl^±9.46 |
| 19 |  |  | 0.7 | 0.90^a-f^±0.03 | | | 289.67^m^±16.35 |
| 20 |  |  | 0.9 | 0.87^b-g^±0.01 | | | 208.40^n^±2.65 |
| 21 |  |  | 1 | 0.22^l^±0.01 | | | 175.66^no^±6.68 |
| 22 | GC2.2 | | 0 | 0.87^a-g^±0.07 | | | 631.83^de^±43.25 |
| 23 |  |  | 0.1 | 0.86^d-g^±0.05 | | | 552.56^fg^±30.87 |
| 24 |  |  | 0.3 | 0.92^a-d^±0.01 | | | 519.32^gh^±15.59 |
| 25 |  |  | 0.5 | 0.72^jk^±0.06 | | | 454.27^ij^±4.09 |
| 26 |  |  | 0.7 | 0.95^ab^±0.02 | | | 382.74^kl^±4.42 |
| 27 |  |  | 0.9 | 0.83^e-h^±0.03 | | | 198.67^no^±4.06 |
| 28 |  |  | 1 | 0.10^m^±1.70 | | | 166.82^no^±3.07 |
| 29 | GC3.1 | | 0 | 0.80^g-j^±0.00 | | | 633.42^de^±22.71 |
| 30 |  |  | 0.1 | 0.82^f-i^±0.03 | | | 524.31^gh^±17.71 |
| 31 |  |  | 0.3 | 0.85^d-g^±0.03 | | | 478.02^hi^±9.46 |
| 32 |  |  | 0.5 | 0.75^ijk^±0.03 | | | 353.09^l^±5.31 |
| 33 |  |  | 0.7 | 0.90^a-f^±0.02 | | | 288.49^m^±12.76 |
| 34 |  |  | 0.9 | 0.75^h-k^±0.05 | | | 177.43^no^±1.53 |
| 35 |  |  | 1 | 0.10^m^±1.70 | | | 153.53^no^±4.05 |

***Note:*** *Values are presented as mean ± standard deviation (SD). Different superscript letters within the same column indicate significant differences among bacterial strains (P < 0.05).*

**Table S4C.** Effects of NaCl concentration on growth (OD₆₀₀) and soluble phosphorus production of phosphate-solubilizing bacteria isolated from wild rice rhizosphere (15 days).

| **No.** | **Strain** | **NaCl concentration (%)** | | | **Evaluation parameters** | | |
| --- | --- | --- | --- | --- | --- | --- | --- |
|  |  |  |  |  | **OD₆₀₀** | **Soluble phosphorus (mg L⁻¹)** | |
| 1 | GC1.2 | | 0 | 0.87^ab^±0.00 | | | 420.35^b^±8.11 |
| 2 |  |  | 0.1 | 0.88^ab^±0.00 | | | 353.24^cd^±37.63 |
| 3 |  |  | 0.3 | 0.63^ghi^±0.02 | | | 291.15^ef^±8.11 |
| 4 |  |  | 0.5 | 0.66^fgh^±0.02 | | | 204.72^i-l^±12.43 |
| 5 |  |  | 0.7 | 0.90^ab^±0.02 | | | 144.24^mno^±0.00 |
| 6 |  |  | 0.9 | 0.66^fgh^±0.01 | | | 102.95^qr^±3.68 |
| 7 |  |  | 1 | 0.21^j^±0.00 | | | 88.20^r^±3.68 |
| 8 | GC1.3 | | 0 | 0.92^a^±0.03 | | | 372.42^c^±9.21 |
| 9 |  |  | 0.1 | 0.85^abc^±0.01 | | | 338.49^cd^±13.27 |
| 10 |  |  | 0.3 | 0.66^fgh^±0.08 | | | 257.22^fg^±8.96 |
| 11 |  |  | 0.5 | 0.76^cde^±0.02 | | | 180.08^klm^±8.11 |
| 12 |  |  | 0.7 | 0.84^abc^±0.02 | | | 134.22^opq^±3.68 |
| 13 |  |  | 0.9 | 0.67^fgh^±0.03 | | | 104.13^qr^±4.45 |
| 14 |  |  | 1 | 0.27^j^±0.00 | | | 89.38^r^±1.77 |
| 15 | GC1.5 | | 0 | 0.86^ab^±0.03 | | | 345.87^cd^±6.76 |
| 16 |  |  | 0.1 | 0.92^ab^±0.03 | | | 295.57^e^±3.54 |
| 17 |  |  | 0.3 | 0.60^hi^±0.00 | | | 227.87^g-j^±22.12 |
| 18 |  |  | 0.5 | 0.65^gh^±0.03 | | | 82.89^r^±15.96 |
| 19 |  |  | 0.7 | 0.88^ab^±0.03 | | | 141.30^nop^±5.11 |
| 20 |  |  | 0.9 | 0.67^fgh^±0.01 | | | 103.54^qr^±1.77 |
| 21 |  |  | 1 | 0.21^j^±0.00 | | | 82.89^r^±2.70 |
| 22 | GC2.2 | | 0 | 0.91^ab^±0.05 | | | 439.82^b^±6.13 |
| 23 |  |  | 0.1 | 0.88^ab^±0.01 | | | 351.77^cd^±8.85 |
| 24 |  |  | 0.3 | 0.60^hi^±0.01 | | | 230.82^ghi^±5.11 |
| 25 |  |  | 0.5 | 0.62^ghi^ ±0.01 | | | 191.74^jkl^±2.04 |
| 26 |  |  | 0.7 | 0.90^ab^±0.02 | | | 215.48^h-k^±3.07 |
| 27 |  |  | 0.9 | 0.67^e-h^±0.01 | | | 104.72^pqr^±4.09 |
| 28 |  |  | 1 | 0.08^k^±0.00 | | | 98.82^qr^±3.68 |
| 29 | GC3.1 | | 0 | 0.89^ab^±0.02 | | | 500.73^a^±25.04 |
| 30 |  |  | 0.1 | 0.83^bcd^±0.03 | | | 332.59^d^±6.76 |
| 31 |  |  | 0.3 | 0.54^i^±0.03 | | | 245.13^gh^±23.94 |
| 32 |  |  | 0.5 | 0.75^def^±0.02 | | | 176.55^lmn^±2.65 |
| 33 |  |  | 0.7 | 0.83^bcd^±0.03 | | | 132.44^opq^±4.45 |
| 34 |  |  | 0.9 | 0.70^efg^±0.03 | | | 109.44^o-r^±1.02 |
| 35 |  |  | 1 | 0.10^k^±1.70 | | | 91.74^r^±1.02 |

***Note:*** *Values are presented as mean ± standard deviation (SD). Different superscript letters within the same column indicate significant differences among bacterial strains (P < 0.05).*

**Table S5A.** Effects of pH on growth (OD₆₀₀) and soluble phosphorus production of phosphate-solubilizing bacteria isolated from wild rice rhizosphere (5 days).

| **No.** | **Strain** | **pH** | | | **Evaluation parameters** | | |
| --- | --- | --- | --- | --- | --- | --- | --- |
|  |  |  |  |  | **OD₆₀₀** | **Soluble phosphorus (mg L⁻¹)** | |
| 1 | GC1.2 | | 3 | 0.32^lm^±0.01 | | | 78.10^g^±5.90 |
| 2 |  |  | 4 | 0.42^hij^±0.04 | | | 165.02^f^±3.03 |
| 3 |  |  | 5 | 0.51^fg^±0.04 | | | 386.71^e^±21.38 |
| 4 |  |  | 6 | 0.71^e^±0.01 | | | 475.80^d^±6.19 |
| 5 |  |  | 7 | 0.97^ab^±0.01 | | | 624.36^b^±12.35 |
| 6 |  |  | 8 | 0.22^no^±0.01 | | | 147.46^f^±9.50 |
| 7 |  |  | 9 | 0.10^p^±0.01 | | | 73.73^g^±4.75 |
| 8 | GC1.3 | | 3 | 0.40^ij^±0.01 | | | 78.71^g^±4.75 |
| 9 |  |  | 4 | 0.48^fgh^±0.01 | | | 157.44^f^±3.12 |
| 10 |  |  | 5 | 0.54^f^±0.02 | | | 403.53^e^±10.96 |
| 11 |  |  | 6 | 0.97^ab^±0.01 | | | 550.17^c^±45.43 |
| 12 |  |  | 7 | 0.82^d^±0.02 | | | 712.59^a^±16.68 |
| 13 |  |  | 8 | 0.21^no^±0.01 | | | 145.51^f^±3.95 |
| 14 |  |  | 9 | 0.10^p^±0.00 | | | 72.75^g^±1.97 |
| 15 | GC1.5 | | 3 | 0.32^klm^±0.02 | | | 75.81^g^±0.61 |
| 16 |  |  | 4 | 0.42^hij^±0.01 | | | 160.86^f^±2.59 |
| 17 |  |  | 5 | 0.64^e^±0.03 | | | 487.17^d^±13.49 |
| 18 |  |  | 6 | 0.83^d^±0.03 | | | 552.74^c^±26.23 |
| 19 |  |  | 7 | 0.92^bc^±0.06 | | | 742.32^a^±13.42 |
| 20 |  |  | 8 | 0.20^no^±0.01 | | | 146.67^f^±3.08 |
| 21 |  |  | 9 | 0.11^p^±0.01 | | | 73.33^g^±1.54 |
| 22 | GC2.2 | | 3 | 0.39^ijk^±0.01 | | | 81.87^g^±2.13 |
| 23 |  |  | 4 | 0.50^fg^±0.01 | | | 164.53^f^±2.22 |
| 24 |  |  | 5 | 0.67^e^±0.03 | | | 484.23^d^±21.85 |
| 25 |  |  | 6 | 0.89^cd^±0.03 | | | 554.39^c^±6.95 |
| 26 |  |  | 7 | 1.01^a^±0.01 | | | 618.19^b^±9.52 |
| 27 |  |  | 8 | 0.25^mn^±0.03 | | | 144.47^f^±4.04 |
| 28 |  |  | 9 | 0.15^op^±0.03 | | | 72.23^g^±2.02 |
| 29 | GC3.1 | | 3 | 0.37^jkl^±0.01 | | | 82.57^g^±1.22 |
| 30 |  |  | 4 | 0.44^ghi^±0.04 | | | 140.25^f^±2.66 |
| 31 |  |  | 5 | 0.69^e^±0.02 | | | 397.41^e^±3.50 |
| 32 |  |  | 6 | 0.93^bc^±0.04 | | | 455.43^d^±46.26 |
| 33 |  |  | 7 | 0.99^ab^±0.03 | | | 569.65^c^±7.72 |
| 34 |  |  | 8 | 0.25^mn^±0.01 | | | 158.54^f^±8.96 |
| 35 |  |  | 9 | 0.15^op^±0.01 | | | 79.27^g^±4.48 |

***Note:*** *Values are presented as mean ± standard deviation (SD). Different superscript letters within the same column indicate significant differences among bacterial strains (P < 0.05).*

**Table S5B.** Effects of pH on growth (OD₆₀₀) and soluble phosphorus production of phosphate-solubilizing bacteria isolated from wild rice rhizosphere (10 days).

| **No.** | **Strain** | **pH** | | | **Evaluation parameters** | | |
| --- | --- | --- | --- | --- | --- | --- | --- |
|  |  |  |  |  | **OD₆₀₀** | **Soluble phosphorus (mg L⁻¹)** | |
| 1 | GC1.2 | | 3 | 0.41^i-l^±0.02 | | | 69.11^j^±2.71 |
| 2 |  |  | 4 | 0.53^gh^±0.02 | | | 238.22^i^±9.41 |
| 3 |  |  | 5 | 0.59^fg^±0.04 | | | 477.63^h^±26.19 |
| 4 |  |  | 6 | 0.88^bc^±0.02 | | | 525.34^gh^±6.87 |
| 5 |  |  | 7 | 0.97^ab^±0.03 | | | 747.12^bc^±10.71 |
| 6 |  |  | 8 | 0.29^mno^±0.01 | | | 244.78^i^±3.87 |
| 7 |  |  | 9 | 0.26^nop^±0.05 | | | 81.59^j^±1.29 |
| 8 | GC1.3 | | 3 | 0.52^ghi^±0.03 | | | 66.79^j^±3.22 |
| 9 |  |  | 4 | 0.64^f^±0.02 | | | 227.30^i^±6.41 |
| 10 |  |  | 5 | 0.65^ef^±0.04 | | | 544.82^fg^±2.14 |
| 11 |  |  | 6 | 0.96^ab^±0.06 | | | 633.05^de^±15.59 |
| 12 |  |  | 7 | 0.97^ab^±0.02 | | | 805.84^ab^±32.18 |
| 13 |  |  | 8 | 0.34^k-n^±0.02 | | | 241.47^i^±12.24 |
| 14 |  |  | 9 | 0.31^l-o^±0.02 | | | 80.49^j^±4.08 |
| 15 | GC1.5 | | 3 | 0.47^hij^±0.02 | | | 66.39^j^±0.97 |
| 16 |  |  | 4 | 0.65^ef^±0.01 | | | 204.59^i^±10.88 |
| 17 |  |  | 5 | 0.75^de^±0.02 | | | 593.05^ef^±4.24 |
| 18 |  |  | 6 | 0.95^ab^±0.04 | | | 682.75^cd^±82.32 |
| 19 |  |  | 7 | 0.98^ab^±0.03 | | | 827.56^a^±6.88 |
| 20 |  |  | 8 | 0.24^op^±0.03 | | | 217.53^i^ ±5.67 |
| 21 |  |  | 9 | 0.17^p^±0.03 | | | 72.51^j^±1.89 |
| 22 | GC2.2 | | 3 | 0.50^ghi^±0.01 | | | 77.32^j^±6.08 |
| 23 |  |  | 4 | 0.75^de^±0.03 | | | 245.05^i^±6.88 |
| 24 |  |  | 5 | 0.96^ab^±0.02 | | | 525.87^gh^±33.07 |
| 25 |  |  | 6 | 0.94^ab^±0.05 | | | 636.84^de^±14.73 |
| 26 |  |  | 7 | 1.01^a^±0.11 | | | 663.03^d^±44.84 |
| 27 |  |  | 8 | 0.39^j-m^±0.01 | | | 221.29^i^±12.82 |
| 28 |  |  | 9 | 0.33^l-o^±0.03 | | | 73.76^j^±4.27 |
| 29 | GC3.1 | | 3 | 0.44^h-k^±0.03 | | | 68.07^j^±0.69 |
| 30 |  |  | 4 | 0.75^de^±0.03 | | | 224.68^i^±6.77 |
| 31 |  |  | 5 | 0.84^cd^±0.01 | | | 584.85^efg^±29.55 |
| 32 |  |  | 6 | 1.00^a^±0.00 | | | 617.52^de^±11.21 |
| 33 |  |  | 7 | 1.02^a^±0.02 | | | 665.56^d^±23.54 |
| 34 |  |  | 8 | 0.38^j-m^±0.03 | | | 246.06^i^±4.77 |
| 35 |  |  | 9 | 0.36^k-n^±0.02 | | | 82.02^j^±1.59 |

***Note:*** *Values are presented as mean ± standard deviation (SD). Different superscript letters within the same column indicate significant differences among bacterial strains (P < 0.05).*

**Table S5C.** Effects of pH on growth (OD₆₀₀) and soluble phosphorus production of phosphate-solubilizing bacteria isolated from wild rice rhizosphere (15 days).

| **No.** | **Strain** | **pH** | | | **Evaluation parameters** | | |
| --- | --- | --- | --- | --- | --- | --- | --- |
|  |  |  |  |  | **OD₆₀₀** | **Soluble phosphorus (mg L⁻¹)** | |
| 1 | GC1.2 | | 3 | 0.67^h-k^±0.02 | | | 61.03^j^±5.65 |
| 2 |  |  | 4 | 0.61^jkl^±0.03 | | | 77.32^ij^±0.61 |
| 3 |  |  | 5 | 0.24^nop^±0.02 | | | 307.18^d^±43.35 |
| 4 |  |  | 6 | 0.98^abc^±0.00 | | | 318.59^d^±9.29 |
| 5 |  |  | 7 | 0.78^efg^±0.03 | | | 431.76^b^±4.13 |
| 6 |  |  | 8 | 0.25^mn^±0.01 | | | 162.51^fg^±5.17 |
| 7 |  |  | 9 | 0.13^q^±0.03 | | | 81.25^hij^±2.58 |
| 8 | GC1.3 | | 3 | 0.77^efg^±0.02 | | | 65.58^j^±1.04 |
| 9 |  |  | 4 | 0.75^fgh^±0.02 | | | 67.14^j^±0.97 |
| 10 |  |  | 5 | 0.85^cde^±0.01 | | | 171.75^efg^±27.16 |
| 11 |  |  | 6 | 0.86^cde^±0.02 | | | 183.87^ef^±5.54 |
| 12 |  |  | 7 | 0.62^l^±0.03 | | | 379.06^c^±3.50 |
| 13 |  |  | 8 | 0.21^n-q^±0.02 | | | 136.27^fg^±9.55 |
| 14 |  |  | 9 | 0.14^opq^±0.01 | | | 68.13^j^±4.77 |
| 15 | GC1.5 | | 3 | 0.63^ijk^±0.02 | | | 53.39^j^±3.28 |
| 16 |  |  | 4 | 0.79^efg^±0.02 | | | 56.91^j^±1.10 |
| 17 |  |  | 5 | 0.60^kl^±0.01 | | | 122.58^ghi^±0.80 |
| 18 |  |  | 6 | 0.94^abc^±0.02 | | | 217.08^e^±55.87 |
| 19 |  |  | 7 | 0.73^f-i^±0.03 | | | 310.26^d^±8.05 |
| 20 |  |  | 8 | 0.30^mn^±0.02 | | | 129.60^gh^±3.22 |
| 21 |  |  | 9 | 0.20^n-q^±0.00 | | | 64.80^j^±1.61 |
| 22 | GC2.2 | | 3 | 0.71^g-j^±0.05 | | | 72.13^ij^±1.26 |
| 23 |  |  | 4 | 0.81^d-g^±0.02 | | | 61.72^j^±2.32 |
| 24 |  |  | 5 | 1.03^a^±0.06 | | | 300.66^d^±21.25 |
| 25 |  |  | 6 | 0.98^ab^±0.08 | | | 392.67^bc^±18.42 |
| 26 |  |  | 7 | 0.71^g-j^±0.01 | | | 416.89^bc^±18.10 |
| 27 |  |  | 8 | 0.22^n-q^±0.01 | | | 155.05^fg^±3.39 |
| 28 |  |  | 9 | 0.14^opq^±0.02 | | | 77.52^ij^±1.70 |
| 29 | GC3.1 | | 3 | 0.63^ijk^±0.03 | | | 70.91^ij^±4.03 |
| 30 |  |  | 4 | 0.82^def^±0.02 | | | 71.06^ij^±2.76 |
| 31 |  |  | 5 | 0.95^abc^±0.05 | | | 378.64^c^±9.89 |
| 32 |  |  | 6 | 0.90^bcd^±0.07 | | | 543.71^a^±33.76 |
| 33 |  |  | 7 | 0.94^abc^±0.05 | | | 525.98^a^±17.94 |
| 34 |  |  | 8 | 0.24^no^±0.02 | | | 162.51^fg^±5.17 |
| 35 |  |  | 9 | 0.13^pq^±0.02 | | | 81.25^hij^±2.58 |

***Note:*** *Values are presented as mean ± standard deviation (SD). Different superscript letters within the same column indicate significant differences among bacterial strains (P < 0.05).*

**Table S6.** Genes associated with environmental stress adaptation identified in the genome of *Enterobacter hormaechei* strain GC1.3.

| **Gene** | **Start position (bp)** | **End position (bp)** | **Strand** | **Predicted function** |
| --- | --- | --- | --- | --- |
| *betA* | 1,254,135 | 1,255,799 | – | Choline dehydrogenase involved in glycine betaine biosynthesis for osmotic stress protection |
| *betB* | 1,255,813 | 1,257,285 | – | Betaine aldehyde dehydrogenase involved in glycine betaine biosynthesis |
| *otsA* | 2,789,536 | 2,790,960 | – | Trehalose-6-phosphate synthase involved in trehalose biosynthesis for osmotic stress tolerance |
| *otsB* | 2,790,935 | 2,791,738 | – | Trehalose-6-phosphate phosphatase involved in trehalose biosynthesis |
| *kdpA* | 1,385,184 | 1,386,863 | – | Membrane component of the high-affinity K⁺ transport system involved in potassium homeostasis under osmotic stress |
| *kdpB* | 1,383,117 | 1,385,165 | – | ATPase component of the high-affinity K⁺ transport system |
| *nhaA* | 751,563 | 752,738 | + | Na⁺/H⁺ antiporter involved in pH homeostasis and ion balance |
| *fur* | 1,369,910 | 1,370,356 | – | Ferric uptake regulator involved in iron homeostasis and oxidative stress response |
| *mntH* | 3,315,712 | 3,316,950 | – | Mn²⁺/Fe²⁺ transporter involved in protection against oxidative stress |

**Figure S1.** Functional map of ABC transporters (ko02010) in the genome of strain *E. hormaechei*.

**Figure S2.** Functional map of the Two-component system (ko02020) in the genome of strain *E. hormaechei*.

Green boxes represent KEGG Orthologs (KOs) annotated based on KofamScan results, indicating the presence of transport systems associated with phosphate and phosphonate.
